# Two Sides to One Story—Aroma Chemical and Sensory Signature of Lugana and Verdicchio Wines

**DOI:** 10.3390/molecules26082127

**Published:** 2021-04-07

**Authors:** Davide Slaghenaufi, Giovanni Luzzini, Jessica Samaniego Solis, Filippo Forte, Maurizio Ugliano

**Affiliations:** Department of Biotechnology, University of Verona, Villa Lebrecht, Via della Pieve 70, 37029 San Pietro in Cariano, Italy; davide.slaghenaufi@univr.it (D.S.); giovanni.luzzini@univr.it (G.L.); jessicaanahi.samaniegosolis@univr.it (J.S.S.); filippo.forte96@gmail.com (F.F.)

**Keywords:** Lugana, Verdicchio, wine aroma, chemical signature, sensory space

## Abstract

Lugana and Verdicchio are two Italian white wines with a Protected Designation of Origin (PDO) label. These two wine types are produced in different regions using the same grape variety. The aim of this work is to investigate the existence of volatile chemical markers that could help to elucidate differences between Lugana and Verdicchio wines both at chemical and sensory levels. Thirteen commercial wine samples were analyzed by Gas Chromatography-Mass Spectrometry (GC-MS), and 76 volatile compounds were identified and quantified. Verdicchio and Lugana had been differentiated on the basis of 19 free and glycosidically bound compounds belonging to the chemical classes of terpenes, benzenoids, higher alcohols, C_6_ alcohols and norisoprenoids. Samples were assessed by means of a sorting task sensory analysis, resulting in two clusters formed. These results suggested the existence of 2 product types with specific sensory spaces that can be related, to a good extend, to Verdicchio and Lugana wines. Cluster 1 was composed of six wines, 4 of which were Lugana, while Cluster 2 was formed of 7 wines, 5 of which were Verdicchio. The first cluster was described as “fruity”, and “fresh/minty”, while the second as “fermentative” and “spicy”. An attempt was made to relate analytical and sensory data, the results showed that damascenone and the sum of 3 of esters the ethyl hexanoate, ethyl octanoate and isoamyl acetate, was characterizing Cluster 1. These results highlighted the primary importance of geographical origin to the volatile composition and perceived aroma of Lugana and Verdicchio wines.

## 1. Introduction

The appellation of origin system is very important for the economic success of local wine on the international markets [1]. A particular added value is recognized for wines from specific geographical areas, which is reflected by the higher price that consumers are willing to pay for these wines [2]. Among the Protected Designation of Origin (PDO) specifications, grape varieties to be employed for winemaking and their area of origin are considered of primary importance.

However, individual varieties are grown in different parts of the world and are used in many PDO production regulations, giving wines with distinctive odor characters [3]. It is well known that grape geographical origin has an impact on wine chemical composition [4,5]. Also volatile compounds like terpenes, norisoprenoids, and fermentation-derived by-products (e.g., esters, alcohols) are affected by grape origin [6,7,8,9,10].

The effect of vineyard site on wines volatile compounds has been shown at various levels, from regional to single-vineyard scale [4,7,11], and the impact on compounds such as terpenes, norisoprenoids, benzenoids and C6 alcohols is well known and somewhat expected [7,11,12,13,14,15,16,17,18]. Other studies indicate an influence of grape origin also on compounds primarily associated with fermentation [10,19,20]. Winemaking techniques also influence wine aroma [21], and grape aroma potential can be managed by applying adapted vinification protocols [3]. The typical odor characteristics of wines from a given PDO arise from complex interaction between grape variety, vineyard location and winemaking [22,23,24]. Combination of these factors lead to a pool of sensory characteristics leading to wine recognition, which can be easier when comparing wines from different varietal origin, while it is more complex when considering wines of the same variety but of different geographical origin.

Lugana and Verdicchio are two Italian PDO white wines. Lugana vineyards are located in the north of Italy, close to the Garda lake in an area spreading across the Veneto and Lombardia regions. Verdicchio is produced in Marche region, in Central Italy. The grapes used for the production of these wines are locally identified with different names, specifically “Trebbiano di Soave” or “Turbiana” for Lugana and “Verdicchio” for Verdicchio. However, although ampelographic descriptions suggested that these could be different grapes, genetic analysis indicated that they are identical varieties [25,26,27]. The different phenotypes observed do not appear indeed to be due to genetic differences, but to different biotypes of the same variety [25]. Few papers investigated the aroma of Verdicchio [28] and Lugana [29,30]. According to Carlin et al. [28], young Verdicchio wines were characterized by fruity, thiolic notes that during aging evolve towards balsamic and anise notes related to the presence of 3-methyl-2,4-nonanedione and methyl salicylate. Among the compounds more relevant for Lugana wine aroma, ethyl esters and norisoprenoids were found to play a major role [29]. Moreover, the varietal thiols 3-mercaptohexan-1-ol (3MH) and 3-mercaptohexyl acetate (3MHA), have been found in Lugana wines above their odor threshold [30]. A marker of this grape variety and so of both Lugana and Verdicchio wines, is methyl salicylate, a benzenoid characterized bybalsamic odors [28]. Lugana and Verdicchio exist on the market as two distinct products associated with individual PDOs. However, no comparison between the aromatic profiles of the two wines has been reported out so far. The question remains therefore open as to whether a chemical and sensory space specific of each one of these two PDOs really exists.

This research aimed at evaluating the existence of aromatic differences between Lugana and Verdicchio wines by means of volatile compounds analysis and sensory evaluation. The wines were collected at local retail shops in the province of Verona between April and June 2019. Volatile compounds were assessed by using multiple extraction techniques such as Solid Phase Extraction (SPE) and Solid Phase Microextraction (SPME) coupled with gas chromatography-mass spectrometry (GC-MS). Odor similarities/diversities between Lugana and Verdicchio were sensorially assessed by means of the sorting task methodology.

## 2. Results and Discussion

### 2.1. Volatile Compounds in Lugana and Verdicchio Wine

A total of 76 volatile compounds have been identified and quantified in wine samples, including 6 alcohols, 4 C_6_ alcohols, 3 acetate esters, 8 ethyl esters, 3 acids, 23 terpenes, 3 sesquiterpenes, 7 norisoprenoids, 8 volatile sulfur compounds (VSC), 10 benzenoids and one further compound, furfural (Table 1). Twenty-four glycosidically-bound compounds were also quantified, 1 higher alcohol, 4 C_6_ alcohols, 7 terpenols, 3 norisoprenoids, and 7 benzenoids.

**Figure 1:**
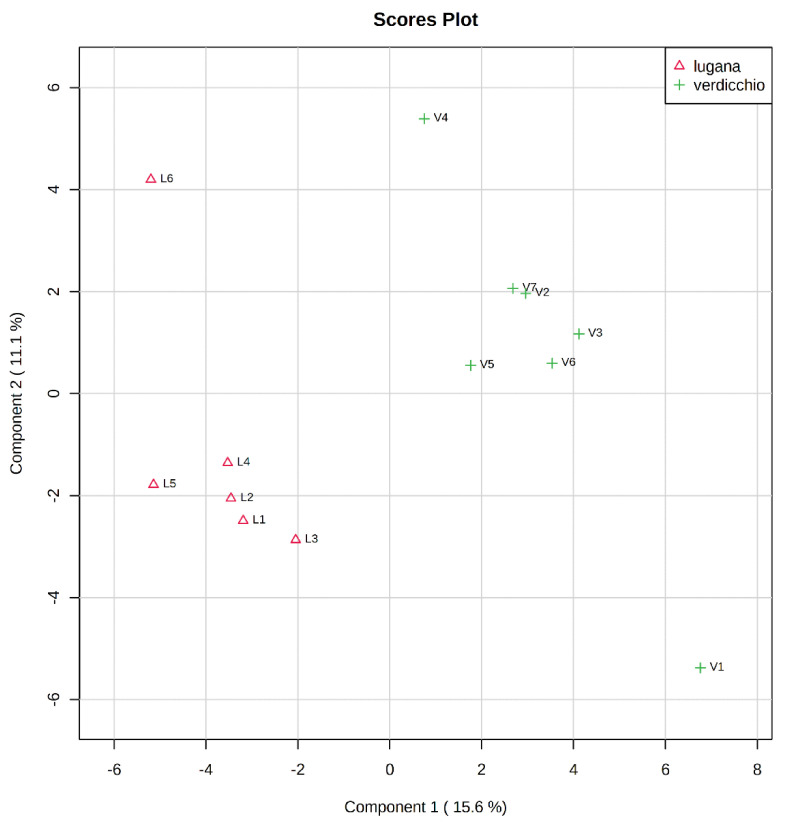
Partial least squares discriminant analysis (PLS-DA) scatterplot of Lugana and Verdicchio wines samples.

Partial least square discriminant analysis (PLS-DA) was used to identify the chemical compounds that differentiated Lugana and Verdicchio wines. The first component (Component 1 15.6%) allowed clear separation between the two wine categories (Figure 1), although the relatively high score obtained for the secondo component (Component 2 11%) suggested significant intra-category variations, probably reflecting differences in grape compositions as well as winemaking techniques. In order to identify the compounds that contribute mostly to discriminate Lugana and Verdicchio wines, Variable Importance for the Projection (VIP) values were obtained from the PLS-DA model. Compounds with a VIP score greater than 1 were considered good candidates for Lugana and Verdicchio discrimination, and they were subjected to Mann-Whitney test with levels of significance of α = 0.1. The compounds with a VIP score > 1 but with a *p* value > 0.1 were not considered as Lugana and Verdicchio marker. Accordingly, a total of 20 parameters (either individual compounds or sum of compounds belonging to the same chemical class) were found to differentiate the two wine categories significantly (Table 2).

Among these, higher content of total terpenes was observed in Lugana wines compared to Verdicchio. The most abundant monoterpene alcohols, namely linalool, α-terpineol, and geraniol, were all detected in significantly higher concentrations in Lugana. Several cyclic terpenes with a *p*-methane structure were also identified in different samples, reaching concentrations of several µg/L such as in the case of terpinen-4-ol and *p*-menthane-1,8-diol. The bi-cyclic terpenes 1,4- and 1,8-cineole were also observed, with the former attaining in some samples concentrations higher than the reported threshold of 0.63 µg/L [31]. These compounds have recently attracted considerable attention for their possible contribution to the eucalyptus and balsamic attributes of red wines [31,32,33], but to our knowledge, their occurrence in white wines is not well documented. Interestingly, with the exclusion of two outliers (L2 and V7), 1,4-cineole as well as the ∑ 1,4-cineole and 1,8-cineole were well correlated with terpinen-4-ol (R^2^ = 0.89 for both correlations if L2 and V7 were excluded), and 1,4-cineole was also correlated with *p*-menthane-1,8-diol (R^2^ = 0.73 if L2 and L6 were excluded). These data confirm the recent observations of Slaghenaufi and Ugliano (2018) [33] concerning the relationship existing between different cyclic terpenes under the acidic conditions of wine, highlighting the possible role of terpinen-4-ol and *p*-menthane-1,8-diol as possible precursors to cineoles, although the presence of some outliers indicate that other precursor might exist. Additionally, a good correlation was also observed between 1,4- and 1,8-cineole (R^2^ = 0.63) across the entire dataset, which is of particular interest considering that these two compounds can act synergistically to favor the expression of hay and eucalyptus attributes [30]. The pool of cyclic terpenes detected could be potentially involved in the balsamic odor notes often perceived in aged samples of these wines [28]. Moreover, terpenes have been reported to have olfactive synergic and additive effects in wine and spirits matrix [34], which should also be considered. A number of different factors determine wine terpene content. Terpenes are produced in grapes through both the 1-deoxy-d-xylulose-5-phosphate/methylerythritol phosphate (DOXP/MEP) pathway and the mevalonic acid (MVA) pathway [35], and in the case of non-aromatic grapes such as Trebbiano they are found mostly in glycosidically bound form, which are then released by yeast during fermentation [36]. The different concentrations of terpenes could be due to viticultural and environmental factors [37,38,39], so that they can be also good markers of geographical wine origin [11].

Lugana wines were characterized by higher concentration of β-damascenone. Cooked apple, quince aroma notes characterize this molecule and its formed mostly during fermentation by acid- or yeast-mediated hydrolysis of multiple precursors [40,41]. Environmental and agronomical factors could influence the precursors level in grapes and consequently, the β-damascenone concentration in wine [7,42,43,44]. The concentration of β-damascenone largely exceed the odor threshold of 0.05 µg/L [45] in all wine samples, contributing to Verdicchio and Lugana aroma. Another norisprenoid differentiating the wines was 3-oxo-α-ionol in the form of glycosidic precursor. During aging, this compound undergoes acid hydrolysis releasing the aglycone moiety [46], as well as acid rearrangements products [33], leading to the formation of megastigmatrienone isomers associated to tobacco aroma in aged wines and spirits [47,48]. The concentration of bound 3-oxo-α-ionol found in Verdicchio wines could be a reservoir of aroma potentially characterizing aged Verdicchio wines.

Occurrence of relatively high levels of free and glycosidically-bound methyl salicylate were also observed, in agreement with recent observations concerning this compound’s high content and its glycosidic precursors in Trebbiano di Soave and Verdicchio wines [28,29,49]. Methyl salicylate is characterized by a distinctive mint, wintergreen aroma [50], and could further contribute to the balsamic character of these wines. Lugana wines showed a significantly higher average concentration (55.6 µg/L) of metyl salicylate compared to Verdicchio wines (21.7 µg/L) with a significance level of *p* = 0.073. Other benzenoids were also detected, with Lugana showing higher content of methyl vanillate and benzyl alcohol, and Verdicchio showing higher concentration in glycoconjugated precursors of vanillyl alcohol, phenylethyl alcohol and 2,6-dimethoxyphenol. Benzenoids are an important class of compounds that could participate to the oaky, spicy and medicinal character of wine [51]. As wines were not stored in barrels, the differences observed are probably related to characteristics of the grapes and/or levels of precursor extraction occurring during winemaking.

The C_6_ alcohols are related to herbaceous odors and they are formed during berry crushing by enzymatic oxidation of grape unsaturated fatty acids [52]. The amount of C_6_ alcohols in wines could be related to different factors like grape variety [53,54], maturity [55], as well as technological factors such as timing of SO_2_ addition [56] and duration of pre-fermentative skin contact [57]. The lower content of *cis*-3-hexenol in Verdicchio wines could be due to the combination of one or more of these factors.

Ubiquitous fermentative compounds contributing to fruity and vinous attributes such as esters, alcohols, and fatty acids were detected and quantified. In some cases, they were found to statistically discriminate the two wine types in the case of methionol and phenylethyl alcohol, occurring at higher concentrations in Verdicchio.

Finally, sulfur compounds and terpenes were two groups of volatiles that appeared to characterize the volatile fraction of Lugana and Verdicchio. Among sulfur compounds, the polyfunctional thiol 3-mercaptohexanol (3-MH), contributing to passionfruit/grapefruit attributes and previously identified among the most potent aroma compounds present in wine [58] was observed. Occurrence of this compound in Lugana wines has been previously reported, in particular in association with reductive winemaking conditions [30]. GC-Olfactometry experiments indicated that certain Verdicchio wines contain 3-MH [28], although quantitative data are not present in the literature. The concentrations observed suggest that this compound contributes to Lugana and Verdicchio’s aroma, although differences observed across the two wine types were not statistically significant. Other sulfur compounds were also detected, some of which rarely reported in white wines, including several disulfides and one trisulfide, typically associated with onion smells. The most abundant of these was carbon disulfide, previously reported in different wines [59].

As the sample set used in the study included wines of different vintages, the possibile influence of vintage was also considered. The volatile compounds influenced by vintage were hexanoic acid (*p*-value = 0.096), *p*-menthane-1,8-diols (*p*-value = 0.093), TPB (*p*-value = 0.078), diethyl sulfide (*p*-value = 0.088), as well as glycosylated *cis*-2-hexen-1-ol (*p*-value = 0.067), and 2,6-dimethoxyphenol (*p*-value = 0.063). None of these compounds was among those responsible for Lugana and Verdicchio differentiation.

### 2.2. Sensory Sorting Task and Relationship between Odor Characteristics and Volatile Composition

**Figure 2:**
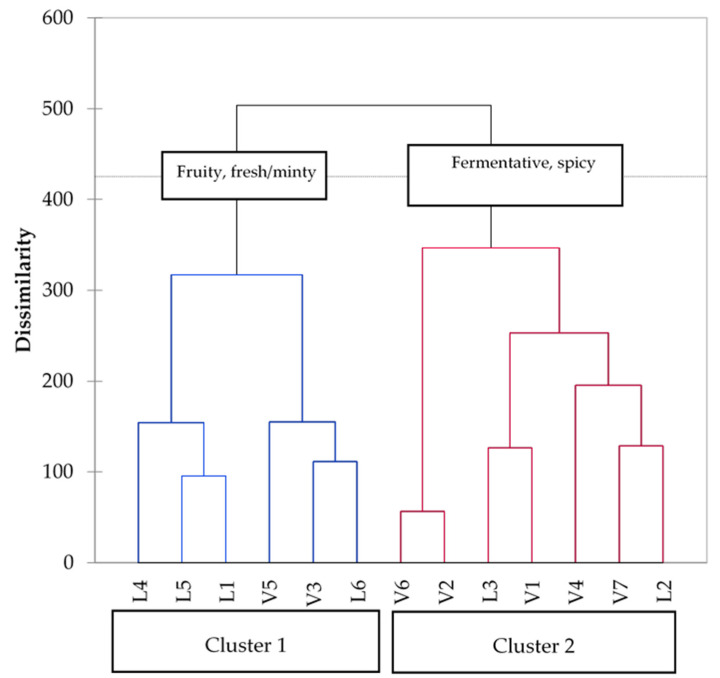
Dendrogram illustrating the results of Lugana and Verdicchio wines sorting task. The dashed line indicates the significance threshold. The blue lines represent **Cluster 1**, the red lines represent **Cluster 2**.

Sensory evaluation of the samples was carried out by means of a sorting task aiming at grouping samples based on their odor similarities. This approach has been successfully used to establish the existence of odor profiles that can be associated to specific variables, including grape variety, yeast strain and wine quality grade [60,61,62]. In the present study, we were interested in clarifying whether two wines with a well-defined product space, such as Verdicchio and Lugana, produced in two different regions of Italy using a single grape variety, also possessed a defined olfactory space. Data obtained from the sorting task analysis were submitted to hierarchical cluster analysis (HCA). Results (Figure 2) showed that wines were divided into two different clusters. Cluster 1 was formed by 6 wines, 4 of which were Lugana, while Cluster 2 was formed by 5 Verdicchio wines and 2 Lugana. Cluster 1 was described mostly as “fruity” and “fresh/minty”, while Cluster 2 was described as “fermentative” and “spicy”. Although odor clusters did not correspond perfectly to wine type, they reflected wine types to a large extent. These observations indicate that a specific olfactory space exists for each one of the two clusters. This could be associated, to a good extent, with one of the two wine types investigated. The notion of a sensory space that can be linked explicitly to individual wine types (e.g., wines from specific varieties, appellations, or geographical origin), has been discussed by a number of authors, in relationship to the concepts of typicality, regional/geographical identity, terroir [63,64,65]. Our data confirm other authors’ findings concerning the existence of specific sensory spaces for single product types, although the presence of outliers reflecting specific viticultural and enological scenarios has to be taken into account [63,66,67].

The volatile compounds characterizing the two clusters were then identified. Twelve volatile compounds showed significant differences (α = 0.1) across the two clusters. Cluster 1 was characterized by a higher concentration of 3-carene, limonene, α-terpineol, and β-damascenone, while Cluster 2 was characterized by 2-butanol, methionol, 3-methyl butanoic acid, terpinolene, geraniol, linalool, ho-trienol, α-terpinen. Moreover, the sum of C_6_ alcohols was found in significantly higher concentration in wines of Cluster 2 (α = 0.1) (Table 3).

The potential impact of these markers on perceived aroma was assessed considering their odor active value (OAV), expressed as the ratio between the concentration and the olfactory threshold of each compound (Table 4).

Compounds with an odor active value higher than 1 (OAV ≥ 1) were considered to have an impact on wine aroma. Ten compounds showed an OAV ≥ 1 in all samples, namely isoamyl alcohol, ethyl butanoate, isoamyl acetate, ethyl hexanoate, ethyl octanoate, 3-methyl butanoic acid, hexanoic acid, octanoic acid, β-damascenone, α-ionone. Moreover, 9 compounds showed an OAV ≥ 1 in at least one sample, namely phenylethyl alcohol, ethyl 3-methyl butanoate, ethyl decanoate, 1,4-cineole, TPB, TDN, 4-ethylguaiacol, methyl salicylate and 3-MH.

Among the compounds with an OAV ≥ 1, only β-damascenone and 3-methyl butanoic acid were significantly different in the two clusters suggesting their contribution to the observed aromatic differences. In particular, β-damascenone may contribute to the fruity attribute of Cluster 1 while the 3-methyl butanoic acid to the fermentative aroma of Cluster 2 wines. Considering that wine aroma is the results from complex interactions between volatile compounds often acting through [68,69], volatile compounds were grouped into aromatic series according to their odor descriptors and chemical family. The series used were fruity, stewed apple/quince, grapefruit, floral, fresh/minty, vinous, spicy, green, reductive, fermentative and evolutive. The series were made in order to obtain a better representation of aroma descriptors commonly used during wine tasting, also considering the aroma families proposed by Ferreira et al., (2010) [70]. The score attributed to each series was calculated as the sum of the average OAV of each volatile compound within that series (Table 4). Significant differences were observed only in three aromatic series: fruity, stewed apple/quince, and floral (Table 5). The stewed apple series included β-damascenone, that as described previously, was significantly higher in Cluster 1. The fruity series was formed by ethyl and acetate esters and it was found to have higher values in Cluster 1. Analyzing the data it can be observed that the concentration, and therefore the OAVs, of the individual esters was not significantly different among clusters, but the sum of esters (and therefore the corresponding score of the aromatic series), was significantly higher in Cluster 1 (*p*-value = 0.063). This indicates that esters, and in particular the most potent ethyl hexanoate, ethyl octanoate and isoamyl acetate, could play a role in the aroma of Cluster 1 wines by a synergic and/or additive effect. These two first series, the fruity and the stewed apple/quince, may explain the fruity notes used by the panel to describe Cluster 1, considering that β-damascenone has been shown to enhance wine perceived fruitiness [70].

The attributes most cited in Cluster 2 were fermentative and spicy. Contrary to what was expected, the aromatic series fermentative, vinous, and spicy were not significantly different between the two clusters. In fact, the aromatic series “vinous” and “fermentative” showed similar values. However, it is however possible that he descriptor “fermentative”, characterizing Cluster 2, was not related to a higher concentration of some compounds, but to a lower content of compounds with a fruity character (esters, norisoprenoid), so that the generic fermentative character was more expressed in Cluster 2 [70].

The aromatic series spicy and fresh/minty were not significantly different among the two clusters. Consequently, it was not possible to determine the group of compounds potentially contributing to the “spicy” and “fresh / minty” sensory attributes, although compounds such as cineoles and methyl salicylate are characterized by minty and balsamic odors. It is also possible that other molecules contributed to this odor note, and they were not measured in the present study, for example, 2,4-methylnonanedione reported in Verdicchio wines by Carlin et al. (2019) [28].

The floral series included terpenes and it was significantly higher in Cluster 1. However, the impact of this series on wine aroma appeared to be low as the score was below 1 in all wine samples. Nevertheless, it is reasonable that this aromatic series was not of primary importance for the characterization of the two aromatic types of wine (Clusters 1 and 2).

Finally, the contribution of the series green and reductive, appeared to be minor due to the low scores and in agreement with the fact that the panel did not use these descriptors to describe wine samples. The aromatic series ‘evolutive’, including TPB and TDN, did not show significant difference among clusters.

## 3. Materials and Methods

### 3.1. Chemicals

Octan-2-ol (97%), 1-hexanol (99%), *cis*-3-hexenol (98%), *trans*-3-hexenol (97%), vanillin (99%), 2,6-dimethoxyphenol (99%), linalool (97%), terpinen-4-ol (≥95%), α-terpineol (90%), nerol (≥97%), geraniol (98%), linalool oxide (≥97%), β-citronellol (95%), *p*-cymene (99%), terpinolene (≥85%), γ-terpinene (≥97%), limonene (97%), 1,8-cineole (99%), 1,4-cineole(≥98.5%), β-damascenone (≥98%), isoamyl alcohol (98%), benzyl alcohol (≥99%), 2-phenylethanol (≥99%), ethyl acetate (99%), ethyl butanoate (99%), ethyl 3-methyl butanoate (≥98%), isoamyl acetate (≥95%), ethyl hexanoate (≥95%), phenylethyl acetate (99%), n-hexyl acetate (≥98%), ethyl lactate (≥98%), ethyl octanoate (≥98%), ethyl decanoate (≥98%), hexanoic acid (≥99%), octanoic acid (≥98%), α-phellandrene (95%), *p*-menthane-1,8-diol (97%), 3-methylbutanoic acid (99%), α-ionone (90%), 1-pentanol (99%), 1-butanol (≥99%), 2-butanol (≥99%), ethyl guaiacol (≥99%), vinyl guaiacol (≥98%), methyl-vanillate (99%), ethyl vanillate (99%), were supplied by Sigma Aldrich (Milan, Italy). Dichloromethane (≥99.8%) and methanol (≥99.8%), were provided by Honeywell (Seelze, Germany). Sodium chloride (≥99.5%) was supplied by Sigma Aldrich (Milan, Italy).

### 3.2. Wine Samples

Thirteen commercial wine samples (6 Lugana and 7 Verdicchio) were purchased for this study. Lugana wines were from 5 different wineries, while Verdicchio wine were all from different wineries. Samples were from 2016, 2017 and 2018 vintages (Table 6). Oenological parameters of the wines are given in Table 6. For each wine, two bottles were acquired and they were pooled before chemical analyses and sensory evaluation took place.

### 3.3. GC-MS Analysis

#### 3.3.1. Volatile Sulfur Compounds Analysis

The low boiling volatile sulfur compounds (VSC) dimethyl sulfide (DMS), diethyl sulfide (DES), Dimethyl disulfide (DMDS), ethyl thioacetate, diethyl disulfide (DEDS), dimethyl trisulfide (DMTS) were analyzed as described by Nguyen et al. (2010) [83] with minor modifications. Ten milliliters of wine were placed in 20 mL vials containing 3 g of NaCl and 100 µL of internal standard (DMS-d6 at 2 mg/L). The samples were incubated at 35 °C for 5 min, then the VSC were extracted by exposing a CAR-PDMS-DVB SPME fiber in the headspace for 30 min. Desorption was performed in the injector at 270 °C for 7 min. Analysis was carried out using a HP 7890A (Agilent Technologies, Santa Clara, CA, USA) gas chromatographer coupled to a 5977B mass spectrometer. Injection was performed in splitless mode. Chromatographic separation was done using a DB-WAX capillary column (30 m × 0.25, 0.25 µm film thickness, Agilent Technologies). Helium was used as carrier gas at 1.0 mL/min of constant flow rate. The oven temperature was programmed starting at 35 °C for 5 min, then raised to 150 °C at 5 °C/min, and finally raised to 240 °C at 10 °C/min and kept for 5 min. Mass spectrometer operated in electron ionization (EI) at 70 eV with ion source temperature at 250 °C and quadrupole temperature at 150 °C. Acquisition was done in Selected Ion Monitoring (SIM), selected ion as well as method performance were reported in Table 7. Calibration curves were obtained using Chemstation software (Agilent Technologies, Inc.) by linear regression, plotting the response ratio (analyte peak area/internal standard peak area) against concentration ratio (analyte added concentration/internal standard concentration).

#### 3.3.2. 3-Mercaptohexanol Analysis

The polyfunctional thiol 3-mercaptohexanol (3-MH) was quantified using ethyl propiolate (ETP) derivatization followed by SPE extraction as described by Herbst-Johnstone et al. (2013) [84]. Internal standard, 1-hexanethiol (100 µL of a solution at concentration 0.25 mg/L in acetonitrile), was added to 50 mL of wine sample together with 1 mL of ethanolic solution of ETP (100 mM). The pH was adjusted to 10.0 ± 0.1 by means of 10 N NaOH additions and stirred for 10 min at room temperature. Then the mixture was centrifugated at 6000 rpm for 10 min. The supernatant was then loaded on a BOND ELUT-ENV, SPE cartridge, containing 1 g of sorbent (Agilent Technlogies, USA), previously activated with 20 mL of methanol and equilibrated with 20 mL of water. The cartridge was then washed with 10 mL of water and the analytes were eluted with 10 mL of dichloromethane. The organic layer was dried with Na_2_SO_4_ and concentrated to about 30 μL under a gentle nitrogen stream. Injection was performed in the injector at 250 °C in splitless mode. Analysis was carried out using a HP 7890A (Agilent Technologies) gas chromatographer coupled to a 5977B mass spectrometer. Chromatographic separation was done using a DB-HeavyWAX capillary column (30 m × 0.25, 0.25 µm film thickness, Agilent Technologies). Helium was used as carrier gas at 1.2 mL/min of constant flow rate. The oven temperature was programmed starting at 70 °C for 5 min, then raised to 162 °C at 10 °C/min, and finally raised to 280 °C at 20 °C/min and kept for 20 min. Mass spectrometer operated in electron ionization (EI) at 70 eV with ion source temperature at 250 °C and quadrupole temperature at 150 °C. Acquisition was done in Selected Ion Monitoring (SIM), selected ion as well as method performance were reported in Table 7. Calibration curves were obtained using Chemstation software (Agilent Technologies, Inc.) by linear regression, plotting the response ratio (analyte peak area/internal standard peak area) against concentration ratio (analyte added concentration/internal standard concentration).

#### 3.3.3. Determination of Major Esters, Alcohols, Acids, Benzenoids, Terpenes and Glycosidically-Bound Compounds

Quantitatively major volatile compounds, mostly of fermentative origin, have been analyzed as described by Slaghenaufi et al. (2019) [11] with minor modifications. Fifty milliliters of sample were added with 20 μL of internal standard solution (2-octanol at 42 mg/L in ethanol) and diluted with 50 mL of distilled water. The solution was then loaded on a BOND ELUT-ENV, SPE cartridge, containing 1 g of sorbent (Agilent Technlogies. USA), previously activated with 20 mL of methanol and equilibrated with 20 mL of water. The cartridge was then washed with 15 mL of water. Free volatile compounds were eluted with 10 mL of dichloromethane, and then concentrated under gentle nitrogen stream to 200 μL prior to GC injection. Bound compounds were recovered with 20 mL of methanol. Methanol was then evaporated under vacuum. Bound compounds were then dissolved in 5 mL of citrate buffer (pH 5). were added to dissolve bound compounds to that 200 μL of an enzyme preparation AR2000 (DSM, Brussels, Belgium, prepared at 70 mg/mL in citrate buffer) were added and incubated at 37 °C for 24 h under shaking (150 rpm).

A calibration curve was prepared for each analyte using seven concentration points and three replicate solutions per point in model wine (12% *v/v* ethanol, 3.5 g/L tartaric acid, pH 3.5). A total of 20 μL of internal standards 2-octanol (42 mg/L in ethanol), was added to the solution. SPE extraction and GC-MS analysis were performed as described above for the samples. Calibration curves were obtained using Chemstation software (Agilent Technologies, Inc.) by linear regression, plotting the response ratio (analyte peak area/internal standard peak area) against concentration ratio (analyte added concentration / internal standard concentration). Method characteristics are reported in Table 7 (Supplementary Materials Figure S1). The 3-oxo-α-ionol analysis was semi-quantitative and they were expressed as µg/L of 2-octanol equivalent (internal standard) as for this compound no commercial standard was available.

#### 3.3.4. Minor Terpenes, Norisoprenoids, Benzenoids

Quantitatively minor terpenes have been analyzed by SPME-GC-MS as described by Slaghenaufi and Ugliano (2018) [33]. Five milliliter of wine added with 5 µL of internal standard solution (octen-2-ol at 420 mg/L in ethanol) was placed into a 20 mL vial, together with 5 mL of mQ water (18.2 MΩ-cm) and 3 g of NaCl. Sample was equilibrated for 1 min at 40 °C. Subsequently SPME extraction was performed using a 50/30 µm divinylbenzene–carboxen–polydimethylsiloxane (DVB/CAR/PDMS) fiber (Supelco, Bellafonte, PA, USA) exposed to sample headspace for 60 min at 40 °C. The fiber was then desorbed into the injector port of a HP 7890A (Agilent Technologies, USA) gas chromatographer coupled to a 5977B mass spectrometer. Injection was performed at 250 °C for 5 min in splitless mode. Chromatographic separation was done using a DB-WAX capillary column (30 m × 0.25, 0.25 µm film thickness, Agilent Technologies, USA). Helium was used as carrier gas at 1.2 mL/min of constant flow rate. The temperature of the GC oven was initially kept at 40 °C for 3 min, and then programmed to raise at 230 °C at 4 °C/min, maintained for 20 min. Mass spectrometer operated in electron ionization (EI) at 70 eV with ion source temperature at 250 °C and quadrupole temperature at 150 °C. Acquisition was done in Selected Ion Monitoring (SIM). Quantification was performed using calibration curve obtained by standards addition at 7 different concentration levels in white wine. A total of 5 μL of internal standards 2-octanol (420 mg/L in Ethanol), 5 mL of water and 3 g of NaCl were added to 5 mL of standard solutions. GC-MS analysis was performed as described above for the samples. Linear term for calibration curves were obtained using Chemstation software (Agilent Technologies, Inc.) by linear regression, plotting the response ratio (analyte peak area/internal standard peak area) against concentration ratio (analyte added concentration/internal standard concentration). The analysis of vitispirane, terpinen-1-ol, TPB, TDN, ho-trienol was semiquantitative as no standards was available. Results for these molecules were expressed as µg/L of 2-octanol equivalent (internal standard) (Table 7).

### 3.4. Sorting Task

A sorting task was used to identify if Verdicchio and Lugana samples were aromatically different. The sorting task was done by an orthonasal evaluation following the method described by Alegre et al. (2017) [61]. The panelists were asked to sort the wines into groups based on aroma similarities, the number of possible groups was not defined. The panel consisted of 16 judges, members of the enology laboratory or enology bachelor students of the University of Verona. The ages of the panelists ranged from 21 to 46 years, and females were 31% of the panel. Twenty milliliters of wine samples were served at room temperature into ISO glasses (ISO 3591:1977) coded with random three-digit numbers. Subsequently, it was asked to indicate two descriptors for each group. Descriptors were chosen among fruity, floral, fresh/minty, green, dry herbs, spicy and fermentative. The similarity matrices of the individual judges were added together to obtain a single global matrix. The global matrix was used to performed hierarchical cluster analysis (HCA) with Ward criteria. To analyze the descriptors, the quotation frequencies of the various descriptors were calculated for each wine. Only terms cited by at least 30% of the panel were considered in the description of the groups [61].

### 3.5. Statistical Analyses

Statistical analysis was performed using XLSTAT 2017 (Addinsoft SARL, Paris, France). Shapiro-Wilk test was used to verify the normal distribution of the data. Due to the small number of samples and their non-normal distribution the Mann-Whitney test was used to determine statistical differences between Lugana and Verdicchio and between the sorting task clusters with a significant level of α = 0.1. PLS-DA was used to identify compounds characterizing Lugana and Verdiccho wines, using MetaboAnalyst v. 5.0 (http://www.metaboanalyst.ca, accessed on 16 March 2021), created at the University of Alberta, Edmonton, AB, Canada [86]. Prior to statistical analysis data were autoscaled.

## 4. Conclusions

The present research investigated the existence of two distinct sensory spaces between two Italian white wines Lugana and Verdicchio, made using the same cultivar Trebbiano di Soave or Verdicchio, but grown in two very different areas. Lugana is produced in the lower Lake Garda and Verdicchio in the Marche region. Despite the limited sampling of 13 wines to our knowledge, this was the first time that these two wines were compared regarding their odor and volatile compound profiles.

Verdicchio and Lugana were similar in the majority of compounds analyzed, showing a general volatile compound profile common to the two wines and probably characteristic of the variety. However, a group of volatile compounds and precursors (13 and 6 respectively) were able to clearly discriminate the two wines. These markers belonged to different chemical classes (terpenes, higher alcohols, benzenoids, norisoprenoids, C_6_ compounds, confirming the existence of chemical signatures associated with the area of production of the wines. Sensory assessment showed that samples were divided into two clusters, highlighting the existence of two types of wine. The first cluster was described as fruity, fresh/minty, mainly formed by the majority of Lugana wines, while the other described with fermentative and spicy notes, with a prevalence of Verdicchio wines. Looking at the chemical bases of the two clusters, it could be shown that the main drivers of diversity for the two clusters were the fatty acid ethyl esters ethyl hexanoate and ethyl octanoate, isoamyl acetate and the norisoprenoid β-damascenone, which appear to well explain the differences in fruitiness. Although the clusters were also distinguished by attributes such as minty and spicy, we were not able to correlate these to any specific chemical feature of the wines analyzed. The limitations of the sensory approach used, namely the sorting task, could be partly responsible for this, as the employed method did not provide quantitative data which can be used to build regression models. Also, applications of even more articulated integrated analytical approaches could lead to identification of additional odorant compounds that could contribute to Verdicchio and Lugana aroma. Finally, we have evidenced the presence, in Lugana and Verdicchio, of a number of cyclic terpenes having minty and balsamic aromas, in particular cineoles. Odor synergies of these compounds and the other balsamic odorant methyl salicylate should be further investigated in relation to the minty character of some of the wines analyzed.

This work contributes to characterize the aroma of Lugana and Verdicchio wines by highlighting their common points and differences. The results obtained highlighted the existence of chemical and sensory differences, highlighting the importance of the geographical origin of the wines as a driver of uniqueness. They will also help winemakers to manage the aroma expression of their wines to enhance the expression of the characteristics mostly associated with the different geographical origin.

## Figures and Tables

**Table 1:** Volatile compounds mean concentration (µg/L) and standard deviation (in brackets) of Lugana and Verdicchio wines.

Sample Code	L1	L2	L3	L4	L5	L6	V1	V2	V3	V4	V5	V6	V7
Variety	Lugana	Lugana	Lugana	Lugana	Lugana	Lugana	Verdicchio	Verdicchio	Verdicchio	Verdicchio	Verdicchio	Verdicchio	Verdicchio
Vintage	2018	2016	2017	2017	2018	2016	2017	2016	2017	2017	2017	2018	2017
Higher alcohols													
1-Butanol	43.3 (2.6)	27.2 (1.9)	16.9 (3.67)	54.4 (4.97)	60 (3.14)	82.4 (4.12)	70.9 (4.54)	93.2 (2.31)	124.5 (2.05)	52.9 (6.87)	82.7 (6.62)	52 (4.68)	58.1 (18.25)
2-Butanol	755 (68)	263 (18)	870 (35)	2014 (121)	1233 (54)	1890 (302)	464 (79)	574 (52)	897 (135)	996 (20)	881 (35)	1848 (185)	861 (34)
Isoamyl alcool	79,262 (1585)	77,943 (6235)	68,735 (6186)	92,012 (14,722)	72,668 (10,900)	111,837 (19012)	85,313 (11,091)	83,035 (2491)	87,285 (10,474)	87,624 (9639)	84,250 (6740)	98,730 (13,822)	80,724 (11,301)
Phenylethyl Alcohol	10,840 (1734)	7162 (215)	4230 (254)	6148 (799)	4225 (422)	5253 (630)	14,404 (1440)	8927 (982)	8457 (507)	9545 (1432)	11,212 (1233)	9083 (1272)	9637 (675)
Methionol	98.1 (10.8)	94.4 (5.7)	84.4 (1.7)	105 (14.7)	68.9 (9.6)	75.4 (9)	174.5 (27.9)	98.8 (16.8)	173.3 (24.3)	156.5 (12.5)	75.3 (11.3)	176.7 (12.4)	191.6 (3.8)
1-Pentanol	151 (21)	191 (29)	144 (13)	127 (13)	171 (14)	108 (8)	140 (17)	146 (19)	212 (28)	133 (15)	160 (24)	166 (12)	174 (10)
∑ Alcohols	91,150 (2351)	85,681 (6239)	74,080 (6191)	100,460 (14,744)	78,426 (10,909)	119,246 (19,025)	100,566 (11,184)	92,874 (2678)	97,148 (10,487)	98,507 (9744)	96,661 (6852)	110,056 (13,882)	91,647 (11,322)
C_6_ alcohols													
1-Hexanol	727 (102)	1027 (175)	694 (62)	720 (79)	665 (100)	1099 (66)	934 (131)	930 (93)	982 (69)	1094 (142)	710 (114)	1029 (93)	1046 (73)
*cis*-3-Hexen-1-ol	22.4 (3.1)	27.9 (0.8)	44.9 (5.4)	28.1 (3.9)	50 (7.5)	45.5 (1.8)	18.6 (1.1)	19.9 (3.2)	20.3 (2.2)	20.9 (3.5)	6.9 (0.8)	29.1 (4.4)	16.2 (2)
*trans*-3-Hexen-1-ol	27.3 (3.8)	65.8 (9.9)	108.5 (13)	47 (1.4)	61.5 (1.8)	70.9 (4.3)	51.6 (6.2)	79.8 (4.8)	80.8 (12.1)	101.2 (7.1)	46.7 (0.9)	50.1 (6.5)	53.1 (3.7)
*cis*-2-Hexen-1-ol	10.2 (1.5)	9.9 (1.4)	11.8 (1.2)	10.1 (1.3)	9 (0.6)	9.3 (0.5)	7.3 (0.7)	10.5 (1.2)	8.7 (0.8)	9.7 (1)	6.5 (0.8)	9.9 (0.4)	7.8 (0.2)
∑ C_6_ alcohols	787 (102)	1131 (175)	859 (64)	805 (79)	786 (100)	1224 (66)	1012 (131)	1040 (93)	1092 (70)	1226 (142)	770 (114)	1118 (93)	1123 (73)
Acetate esters													
Isoamyl acetate	952 (67)	102 (2)	857 (120)	1262 (88)	1646 (115)	294 (18)	484 (53)	228 (11)	340 (58)	388 (23)	766 (54)	1056 (148)	356 (28)
*n*-Hexyl acetate	114.5 (11.5)	1.71 (0.2)	40.1 (1.2)	56.8 (4)	57.8 (2.3)	32.7 (0.7)	37.1 (5.2)	12.6 (1.3)	16.8 (2.2)	37.3 (1.9)	26.3 (0.8)	63.6 (6.4)	33.1 (5.3)
2-Phenethyl acetate	298.6 (50.8)	26.9 (3.2)	63.5 (1.3)	82.2 (8.2)	82.7 (14.1)	24.5 (1.2)	134.7 (22.9)	45.8 (2.3)	47.7 (1.9)	94.1 (16)	134 (9.4)	88.9 (10.7)	87.6 (12.3)
∑ Acetate esters	1365 (85)	131 (4)	961 (120)	1401 (89)	1786 (116)	351 (18)	656 (58)	286 (12)	405 (58)	520 (28)	927 (54)	1208 (148)	477 (31)
Ethyl esters													
Ethyl butanoate	254 (2)	134 (1)	248 (7)	250 (20)	253 (40)	333 (30)	232 (3)	279 (12)	209 (8)	297 (7)	327 (14)	333 (17)	132 (3)
Ethyl 2-methylbutyrate	3.08 (0.15)	1.18 (0.18)	3.19 (0.41)	6.91 (0.62)	1.94 (0.27)	9.49 (1.33)	4.29 (0.69)	5.59 (0.84)	14.53 (0.73)	1.38 (0.17)	5.65 (0.34)	3.09 (0.09)	4.02 (0.2)
Ethyl 3-methylbutanoate	5.34 (0.69)	24.27 (3.4)	16.45 (0.82)	12.4 (1.12)	4.43 (0.31)	15.58 (1.71)	9.85 (0.49)	3.33 (0.07)	23.07 (2.54)	2.62 (0.39)	11.11 (1.44)	10.36 (0.93)	6.95 (0.14)
Ethyl 3-hydroxybutyrate	182 (20)	122 (7)	145 (7)	125 (4)	102 (6)	101 (6)	115 (5)	111 (2)	107 (12)	144 (17)	138 (22)	96 (16)	99 (9)
Ethyl hexanoate	642 (71)	260 (16)	779 (55)	754 (23)	736 (88)	845 (93)	551 (50)	668 (100)	549 (33)	538 (32)	677 (115)	705 (99)	567 (85)
Ethyl octanoate	990 (129)	435 (26)	648 (26)	591 (35)	740 (81)	894 (116)	638 (64)	801 (96)	365 (7)	556 (94)	769 (131)	652 (104)	562 (96)
Ethyl decanoate	223 (4)	52 (7)	124 (12)	84 (10)	120 (11)	120 (7)	117 (5)	210 (13)	60 (7)	89 (7)	120 (8)	141 (24)	95 (13)
Ethyl lactate	1157 (197)	2073 (187)	3774 (75)	14,745 (1327)	2258 (203)	4712 (236)	4301 (473)	11,886 (951)	13,673 (1641)	8512 (340)	9328 (1492)	1354 (190)	3460 (346)
∑ Ethyl esters	3455 (246)	3102 (189)	5738 (98)	16,568 (1328)	4215 (240)	7030 (280)	5968 (480)	13,965 (961)	15,001 (1641)	10,140 (355)	11375 (1503)	3294 (240)	4926 (369)
Acids													
3-Methylbutanoic acid	247 (20)	259 (5)	162 (23)	205 (6)	163 (8)	124 (7)	305 (12)	193 (8)	188 (21)	235 (5)	177 (18)	226 (34)	278 (11)
Hexanoic acid	6282 (691)	5655 (735)	4171 (459)	4222 (253)	4806 (336)	6640 (133)	4406 (441)	5035 (705)	3397 (272)	5385 (162)	4245 (340)	4083 (286)	5305 (849)
Octanoic acid	10,053 (1709)	8636 (691)	8437 (759)	7569 (454)	8407 (1093)	9318 (1025)	8452 (1183)	9137 (365)	6767 (1083)	8385 (1258)	8233 (1153)	7986 (479)	8596 (1375)
∑ Acids	16,581 (1843)	14,551 (1009)	12,770 (887)	11,997 (520)	13,377 (1144)	16,082 (1034)	13,163 (1263)	14,365 (794)	10,351 (1116)	14,005 (1268)	12,655 (1202)	12,294 (559)	14,179 (1616)
Terpenes													
1,4-Cineole	0.28 (0.03)	0.72 (0.03)	0.54 (0.06)	0.41 (0.01)	0.33 (0.03)	1.06 (0)	0.39 (0.05)	0.81 (0.04)	0.89 (0)	0.62 (0)	0.62 (0.08)	0.34 (0.01)	0.59 (0.01)
Limonene	0.83 (0.12)	0.35 (0.04)	0.24 (0)	0.44 (0.07)	0.51 (0.11)	0.54 (0)	0.08 (0.01)	0.26 (0.06)	0.38 (0)	0.51 (0)	0.33 (0)	0.32 (0.02)	0.29 (0.01)
1,8-Cineole	0.07 (0.02)	0.09 (0.01)	0.12 (0.08)	0.03 (0.01)	0.1 (0)	0.46 (0)	0.1 (0.01)	0.21 (0.06)	0.22 (0)	0.27 (0)	0.19 (0.04)	0.11 (0.04)	0.08 (0.01)
γ-Terpinen	0.13 (0.01)	3.05 (0.14)	<0.03	1.96 (0.06)	3.58 (0.69)	0.65 (0)	0.16 (0.02)	<0.1	4.29 (0)	5.13 (0)	0.17 (0.11)	1.1 (1.46)	1.29 (1.65)
*p*-Cymene	0.43 (0.01)	0.35 (0.06)	0.41 (0.08)	0.57 (0.05)	0.58 (0.04)	0.74 (0)	<0.24	0.55 (0.11)	0.35 (0)	0.52 (0)	0.51 (0.03)	0.5 (0.01)	0.59 (0.06)
Terpinolene	0.33 (0)	0.13 (0.01)	0.15 (0)	0.17 (0.01)	0.23 (0.02)	0.22 (0)	<0.09	0.11 (0.01)	0.18 (0)	0.25 (0)	0.13 (0)	0.15 (0.01)	0.17 (0.04)
Linalool	4.95 (0.42)	0.06 (0.03)	0.29 (0.11)	1.18 (0.58)	1.63 (0.61)	0.9 (0)	<0.03	0.69 (0.32)	<0.03	<0.03	0.33 (0.18)	0.53 (0.33)	0.51 (0.06)
Terpinen-1-ol	0.02 (0)	4.85 (1.94)	0.02 (0.01)	3.6 (0.64)	1.52 (1.54)	8.42 (0)	0.01 (0.01)	4.9 (0.81)	0.07 (0)	0.12 (0)	0.06 (0.06)	1.9 (0.11)	3.35 (0.88)
Terpinen-4-ol	0.64 (0.09)	0.48 (0.08)	0.9 (0.17)	0.77 (0.06)	0.73 (0.13)	1.24 (0)	0.75 (0.09)	1.08 (0.01)	1.02 (0)	0.88 (0)	1.01 (0.01)	0.84 (0.14)	0.39 (0.52)
Ho-trienol	nd	0.02 (0.02)	0.03 (0.02)	0.23 (0.01)	nd	nd	0.76 (0.01)	0.07 (0.01)	nd	0.02 (0)	nd	nd	0.03 (0)
α-Terpineol	3.76 (0.28)	2.35 (0.17)	1.83 (0.54)	2.68 (0.32)	3.01 (0.25)	3.29 (0)	1.48 (0.18)	1.9 (0.36)	2.99 (0)	2.44 (0)	1.88 (0.11)	1.57 (0.11)	0.86 (0.83)
β-Citronellol	2.58 (0.2)	0.32 (0.24)	3.46 (0.31)	0.81 (0.37)	2.46 (2.23)	0.1 (0)	1.25 (0.15)	0.03 (0.01)	0.03 (0)	1.78 (0)	2.35 (0.09)	0.34 (0.15)	0.03 (0.01)
Nerol	0.88 (0.53)	3.79 (0.09)	3.15 (0.3)	0.11 (0.1)	0.2 (0.04)	0.18 (0)	6.82 (0.82)	0.08 (0.01)	0.45 (0)	0.79 (0)	3.69 (1.03)	0.09 (0.04)	0.1 (0)
Geraniol	2.19 (0.76)	1.46 (0.21)	0.14 (0.11)	1.25 (0.64)	2.73 (1.7)	0.52 (0)	0.09 (0.01)	1.09 (0.74)	0.78 (0)	0.47 (0)	0.31 (0.06)	0.19 (0.04)	0.49 (0.12)
3-Carene	0.41 (0.04)	0.04 (0.06)	0.07 (0.08)	0.28 (0.03)	0.46 (0.11)	0.23 (0)	0 (0)	0.07 (0.08)	0.07 (0)	0.09 (0)	0.15 (0.01)	0.28 (0.02)	0.09 (0.11)
α-Phellandrene	0.4 (0)	0.09 (0.09)	0.18 (0.2)	0.2 (0.26)	0.51 (0.06)	<0.03	0.11 (0.01)	0.21 (0.28)	0.06 (0)	0.03 (0)	0.27 (0.36)	0.39 (0.06)	0.44 (0.03)
α-Terpinen	0.1 (0.01)	0.05 (0.06)	<0.03	<0.03	<0.03	<0.03	<0.03	<0.03	0.1 (0)	<0.03	0.04 (0.05)	<0.03	<0.03
β-Myrcene	0.02 (0.01)	0.01 (0.01)	0.01 (0.01)	0.01 (0)	0.01 (0.01)	0.01 (0)	0.01 (0.01)	0.01 (0.01)	0.01 (0)	0.1 (0)	0.01 (0)	0.01 (0)	0.01 (0)
*cis*-Linalooloxide	1.5 (0.17)	9.1 (1.09)	2.32 (0.32)	3.4 (0.31)	1.84 (0.31)	4.03 (0.6)	2.66 (0.27)	0.26 (0.03)	4.44 (0.18)	2.87 (0.46)	2.26 (0.38)	1.07 (0.12)	2.52 (0.43)
*trans*-Linalooloxide	1.45 (0.07)	6.54 (0.78)	2.04 (0.27)	4.54 (0.09)	5.52 (0.61)	5.12 (0.61)	2.24 (0.16)	2.29 (0.39)	2.76 (0.19)	1.11 (0.07)	2 (0.24)	2.22 (0.24)	2.43 (0.22)
*p*-Menthane-1-8-diol	<0.03	7.85 (0.28)	0.14 (0.02)	<0.03	0.56 (0.1)	1.78 (0.05)	0.32 (0.03)	3.11 (0.53)	2.99 (0.18)	2.35 (0.31)	0.36 (0.05)	<0.03	2.06 (0.29)
∑ Terpenes	21.4 (1.1)	43.6 (2.42)	17.2 (0.89)	23.6 (1.27)	27.2 (3.41)	31.5 (0.86)	18 (0.92)	18.4 (1.65)	23.5 (0.32)	21.7 (0.56)	17.7 (1.21)	12.5 (1.55)	17.1 (2.19)
Sesquiterpenes													
Nerolidol 1	40.7 (0.01)	23 (1.12)	<0.05	31.4 (3.49)	<0.05	51.7 (0)	0.3 (0.04)	38 (0.09)	25.5 (0)	40 (0)	<0.05	26.4 (2.76)	30 (1.11)
Bisabolol	0.59 (0.01)	0.59 (0.01)	0.6 (0.03)	0.6 (0.01)	0.64 (0.05)	0.59 (0)	0.29 (0.04)	0.6 (0)	0.6 (0)	0.6 (0)	0.59 (0.01)	0.58 (0)	0.59 (0)
Farnesol 1	0.03 (0.01)	0.03 (0.01)	0.03 (0.01)	0.06 (0.01)	0.02 (0.01)	0.03 (0)	0.01 (0.01)	0.02 (0.01)	0.04 (0)	0.02 (0)	0.02 (0.01)	0.03 (0)	0.05 (0.07)
∑ Sesquiterpenes	41.3 (0.02)	23.6 (1.12)	0.6 (0.03)	32 (3.49)	0.7 (0.05)	52.3 (0)	0.6 (0.05)	38.6 (0.09)	26.2 (0)	40.6 (0)	0.6 (0.01)	27 (2.76)	30.7 (1.11)
Norisoprenoids													
β-Damascenone	0.91 (0.07)	0.51 (0)	0.51 (0.1)	1.77 (0.28)	0.89 (0.13)	0.64 (0)	0.3 (0.04)	0.48 (0.02)	0.36 (0)	0.43 (0)	1.13 (0.01)	0.31 (0.02)	0.33 (0.01)
Vitispirane 1	0.25 (0.01)	1.04 (0.08)	0.64 (0.09)	0.61 (0.04)	0.44 (0.08)	1.38 (0)	0.32 (0.04)	nd	0.81 (0)	0.77 (0)	0.65 (0.07)	0.38 (0)	0.51 (0.06)
Vitispirane 2	0.21 (0.01)	0.9 (0.05)	0.55 (0.13)	0.34 (0.15)	0.29 (0.04)	0.63 (0)	0.24 (0.03)	0.61 (0.86)	0.56 (0)	0.51 (0)	0.45 (0.01)	0.19 (0.02)	0.29 (0.04)
TPB	0.05 (0.01)	0.09 (0)	0.09 (0.01)	0.1 (0.01)	0.06 (0.01)	0.01 (0)	0.06 (0.01)	0.11 (0.01)	0.07 (0)	0.07 (0)	0.12 (0.01)	0.05 (0.01)	0.07 (0)
TDN	0.89 (0.08)	3.12 (0.21)	2.74 (0.56)	1.51 (0.04)	1.63 (0.4)	0.14 (0)	1.3 (0.16)	1.66 (0.76)	2.13 (0)	2.07 (0)	1.71 (0.04)	0.95 (0.06)	1.59 (0.31)
α-Ionone	0.8 (0.02)	0.76 (0.05)	0.75 (0.06)	0.77 (0.09)	0.76 (0.07)	0.77 (0.06)	0.76 (0.03)	0.75 (0.13)	0.75 (0.04)	0.77 (0.1)	0.76 (0.11)	0.75 (0.12)	0.75 (0.08)
α-Ionol	0.76 (0.04)	0.77 (0.02)	0.75 (0.04)	0.74 (0.05)	0.75 (0.03)	0.81 (0.11)	0.76 (0.04)	0.75 (0.05)	0.75 (0.04)	0.76 (0.08)	0.75 (0.03)	0.75 (0.05)	0.79 (0.06)
3-Hydroxy-β-damascone	nd	0.01 (0)	nd	0.01 (0)	nd	0.01 (0)	nd	0.01 (0)	0.09 (0.01)	0.01 (0)	nd	nd	0.01 (0)
3-Oxo-α-ionol	2.5 (0.1)	3.11 (0.06)	3.36 (0.17)	2.01 (0.14)	4.83 (0.58)	5.98 (0.66)	3.74 (0.56)	2.27 (0.2)	4.14 (0.7)	8.14 (0.16)	1.24 (0.07)	5.5 (0.61)	4.31 (0.73)
∑ Norisoprenoids	5.9 (0.15)	8.37 (0.22)	8.19 (0.6)	6.9 (0.33)	8.91 (0.72)	8.35 (0.67)	6.91 (0.58)	6.03 (0.8)	8.29 (0.7)	12.25 (0.21)	5.7 (0.14)	8.3 (0.63)	7.85 (0.8)
Volatile sulfur compounds (VSC)													
3-MH	0.12 (0.01)	0.3 (0.01)	0.29 (0.04)	<0.05	<0.05	0.12 (0.01)	0.15 (0.02)	<0.05	0.36 (0.06)	<0.05	<0.05	0.2 (0.02)	<0.05
Carbon disulfide	128.7 (18.3)	50.2 (13.6)	73.5 (24.1)	87.2 (9.2)	54.1 (1.9)	92.7 (25.3)	88 (7.2)	115.4 (12.5)	75.8 (6.2)	98.4 (0.1)	92.9 (3.4)	100.4 (6.8)	123.8 (0.5)
DMS	1.69 (0.04)	1.7 (0.04)	1.46 (0.07)	1.06 (0.03)	0.98 (0.02)	1.96 (0.08)	0.21 (0.03)	3.1 (0.02)	1.66 (0.01)	1.4 (0.04)	0.38 (0.01)	0.69 (0.01)	1.16 (0.02)
DES	1.18 (0.06)	0.38 (0.01)	0.69 (0.03)	0.69 (0.06)	0.63 (0.05)	0.72 (0.22)	0.6 (0.13)	1.1 (0.06)	0.79 (0)	0.55 (0.01)	0.67 (0.01)	0.86 (0.09)	0.77 (0.18)
DMDS	<0.09	<0.09	<0.09	<0.09	<0.09	<0.09	<0.09	<0.09	<0.09	<0.09	<0.09	<0.09	<0.09
Ethyl thioacetate	1.07 (0)	0.76 (0.26)	0.56 (0.1)	0.81 (0.04)	0.63 (0.3)	1.48 (1.74)	0.86 (0.4)	0.69 (0.01)	0.6 (0.33)	0.36 (0.03)	0.7 (0.1)	1.03 (0.07)	0.98 (0.3)
DEDS	<0.18	<0.18	<0.18	<0.18	<0.18	<0.18	<0.18	<0.18	<0.18	<0.18	<0.18	<0.18	<0.18
DMTS	<0.21	<0.21	<0.21	<0.21	<0.21	<0.21	<0.21	<0.21	<0.21	<0.21	<0.21	<0.21	<0.21
∑ VSC	132.8 (18.3)	53.4 (13.6)	76.5 (24.1)	89.8 (9.2)	56.5 (1.9)	97 (25.3)	89.9 (7.2)	120.4 (12.5)	79.3 (6.2)	100.8 (0.1)	94.6 (3.4)	103.3 (6.8)	126.8 (0.6)
Benzenoids													
Methyl salicylate	19 (1.13)	37.7 (0.2)	20.9 (3.44)	192 (16.24)	26.7 (4.5)	49.8 (0)	7.6 (0.91)	17.4 (0.62)	13.8 (0)	26.5 (0)	19.1 (0.85)	56.4 (2.4)	11.4 (0.69)
4-Ethyl guaiacol	3.19 (0.51)	3.3 (0.56)	3.01 (0.48)	3.39 (0.51)	2.99 (0.18)	3.07 (0.09)	18.47 (3.9)	0 (0)	103.2 (12.39)	0 (0)	0 (0)	3.07 (0.46)	3.43 (0.58)
4-Ethyl phenol	3.44 (0.1)	3.47 (0.59)	3.46 (0.28)	3.44 (0.14)	3.43 (0.58)	3.56 (0.28)	3.45 (0.1)	3.44 (0.55)	3.46 (0.28)	3.44 (0.48)	3.47 (0.59)	3.45 (0.17)	3.44 (0.24)
2,6-Dimethoxyphenol	3.01 (0.09)	3.05 (0.31)	3.01 (0.33)	3.22 (0.29)	3.02 (0.15)	3.04 (0.18)	4.62 (0.14)	3.03 (0.15)	5.11 (0.36)	3.01 (0.21)	3.03 (0.27)	3.02 (0.27)	3.02 (0.51)
Vanillin	<0.2	6.31 (1.01)	4.33 (0.17)	10.54 (1.05)	6.66 (0.73)	<0.2	18.89 (0.38)	2.26 (0.18)	3.87 (0.12)	4.59 (0.6)	10.47 (1.05)	4.97 (0.5)	3.59 (0.29)
Methyl vanillate	7.77 (0.47)	10.83 (0.22)	7.79 (1.32)	6.75 (0.61)	8.69 (0.87)	10.56 (0.42)	6.1 (0.49)	10.4 (0.62)	6.52 (0.65)	6.61 (0.93)	6.83 (0.41)	7.27 (0.87)	5.57 (0.95)
Ethyl vanillate	2.25 (0.36)	7.3 (0.73)	3.34 (0.57)	2 (0.14)	10.07 (1.71)	8.68 (1.3)	1.83 (0.15)	2.47 (0.12)	2.47 (0.07)	3.68 (0.07)	1.92 (0.19)	5.2 (0.62)	2.59 (0.44)
Vanillyl alcohol	1.3 (0.18)	1.35 (0.23)	4.32 (0.3)	7.49 (0.75)	4.8 (0.82)	11.37 (1.14)	8.22 (1.32)	1.49 (0.13)	6.93 (1.18)	1.39 (0.14)	1.26 (0.05)	8.79 (0.18)	1.48 (0.18)
Benzaldehyde	17.1 (1)	16.2 (0.8)	19.8 (1.6)	59.8 (9)	34.3 (2.7)	19.1 (1.1)	16.7 (0.8)	25.6 (1.8)	26.3 (1.3)	16.2 (1)	15.6 (1.7)	17.8 (2.5)	14.8 (1.3)
Benzyl Alcohol	147 (15)	188 (6)	95 (12)	359 (61)	194 (10)	350 (59)	73 (12)	157 (19)	201 (30)	109 (18)	72 (3)	165 (28)	81 (2)
∑ Benzenoids	204 (15)	278 (6)	165 (13)	647 (64)	294 (11)	459 (59)	159 (12)	223 (19)	373 (33)	175 (18)	134 (4)	275 (28)	130 (3)
Furfural	2.77 (0.19)	12.77 (2.17)	14.69 (1.76)	20.07 (1)	11.26 (1.13)	69.44 (5.56)	16.04 (1.44)	69.6 (3.48)	58.44 (1.75)	17.58 (2.11)	8.96 (0.9)	16.08 (1.29)	14.94 (1.79)
Glycosidically bound compounds													
1-Hexanol	11.3 (0.45)	20.8 (0.83)	9.7 (1.65)	13.7 (1.51)	14.3 (2)	39.5 (2.77)	2.9 (0.32)	12.5 (2)	23.2 (4)	22.1 (3.53)	16.5 (0.83)	19.4 (3.11)	11.9 (0.71)
*cis*-3-Hexen-1-ol	0.05 (0)	0.04 (0.01)	0.26 (0.02)	0.04 (0)	0.06 (0)	0.43 (0.06)	0.17 (0.02)	0.42 (0.05)	0.05 (0.01)	0.09 (0.01)	0.04 (0)	0.18 (0.01)	0.06 (0.01)
*trans*-3-Hexen-1-ol	0.75 (0.03)	4.32 (0.52)	2.39 (0.33)	2.06 (0.25)	3.12 (0.28)	5.99 (0.12)	0.42 (0.04)	1.07 (0.07)	6.18 (1.07)	6.02 (0.84)	0.12 (0.01)	0.99 (0.08)	2.26 (0.32)
*cis*-2-Hexen-1-ol	2.51 (0.05)	13.62 (1.5)	13.11 (1.05)	13.78 (0.55)	13.54 (1.08)	12.91 (1.94)	6.03 (0.72)	1.17 (0.13)	12.16 (2.1)	13.66 (1.09)	14.97 (1.5)	0.5 (0.03)	14.18 (0.85)
*cis*-Linalool oxide	4.87 (0.58)	6.94 (0.28)	5.7 (0.63)	1.52 (0.2)	6.63 (0.4)	4.53 (0.09)	0.97 (0.16)	5.94 (0.59)	1.24 (0.21)	3.85 (0.19)	0.11 (0.02)	0.76 (0.02)	0.52 (0.09)
*trans*-Linalool oxide	5.23 (0.31)	10.51 (0.53)	7.47 (0.22)	1.81 (0.31)	9.5 (0.48)	4.97 (0.8)	0.97 (0.14)	10.87 (1.63)	3.02 (0.52)	5.98 (0.66)	0.09 (0.01)	0.79 (0.07)	0.16 (0.01)
Linalool	12.21 (0.61)	0.04 (0)	0.1 (0)	0.81 (0.05)	1.92 (0.17)	0.08 (0)	2.13 (0.17)	0.55 (0.04)	3.02 (0.52)	2.39 (0.05)	31.53 (2.21)	0.48 (0.01)	0.09 (0.01)
α-Terpineol	25.1 (0.8)	6.2 (0.3)	17 (2.4)	3.5 (0.5)	31.1 (3.7)	6.2 (0.7)	17.2 (2.2)	26.8 (4)	19.2 (3.3)	16.1 (1.6)	13 (0.8)	68.9 (2.8)	27.9 (3.1)
β-Citronellol	34.5 (4.8)	1.9 (0.1)	5.5 (0.6)	1 (0.1)	17.2 (1.2)	1.3 (0.1)	50.3 (4)	1.2 (0.1)	4.1 (0.7)	3.7 (0.4)	32.1 (5.5)	7.1 (0.9)	5 (0.1)
Methyl salicylate	229 (23)	317 (25)	142 (16)	132 (21)	238 (19)	355 (53)	93 (14)	62 (6)	19 (3)	172 (12)	149 (25)	472 (47)	165 (20)
Nerol	11.3 (1.5)	25.8 (1)	17.2 (2.2)	8.5 (0.4)	14.2 (14.6)	4.3 (0.3)	5.6 (0.6)	4.7 (0.1)	24.3 (4.2)	16.3 (2.1)	17.1 (2.6)	9.9 (9.5)	14.3 (2.1)
Geraniol	19.3 (1.6)	60.1 (4.8)	41.6 (2.5)	12.6 (0.4)	22.9 (45.3)	21.8 (1.1)	28.5 (368.9)	100 (7)	28.2 (35.9)	29 (4.4)	24.8 (4)	232.5 (37.2)	55.5 (6.7)
Benzyl Alcohol	7189 (863)	2474 (198)	2510 (326)	622 (12)	6711 (671)	988 (168)	8150 (245)	5424 (271)	1848 (319)	1628 (195)	2143 (214)	10,893 (436)	2871 (287)
α-Ionol	1.03 (0.15)	0.8 (0.07)	0.79 (0.1)	0.74 (0.1)	0.97 (0.04)	0.76 (0.13)	1.8 (0.16)	1.24 (0.17)	1.02 (0.18)	0.76 (0.05)	0.86 (0.08)	1.57 (0.25)	0.79 (0.08)
Phenylethyl Alcohol	1796 (198)	692 (111)	919 (138)	190 (6)	1599 (64)	254 (8)	4020 (80)	2605 (234)	6620 (142)	681 (75)	995 (60)	3219 (64)	1572 (236)
2,6-Dimethoxyphenol	3.15 (0.5)	3.05 (0.31)	3.05 (0.27)	3.01 (0.51)	3.09 (0.4)	3.04 (0.18)	3.47 (0.52)	3.6 (0.14)	3.75 (0.65)	3.1 (0.06)	3.12 (0.31)	3.78 (0.3)	3.1 (0.47)
3-Hydroxy-β-damascone	2.62 (0.42)	0.71 (0.08)	1.21 (0.19)	0.22 (0.01)	1.97 (0.04)	0.23 (0.04)	0.72 (0.02)	4.76 (0.76)	3.17 (0.55)	0.74 (0.01)	1.02 (0.07)	4.3 (0.56)	1.77 (0.19)
Vanillin	18.1 (2.4)	2.4 (0.4)	6.3 (1.1)	4.7 (0.6)	9.3 (1.3)	2.3 (0.1)	24.5 (4.2)	4.9 (0.2)	5 (0.9)	2.3 (0.2)	10 (1)	4.5 (0.5)	21.5 (1.5)
Methyl vanillate	42.7 (4.7)	19.7 (3.2)	23.9 (2.2)	4.6 (0.1)	34.7 (3.1)	7.8 (0.4)	108.2 (15.2)	150.3 (12)	24.8 (4.3)	13.5 (0.8)	21.1 (2.5)	92.4 (2.8)	26.5 (1.3)
Ethyl vanillate	26.9 (4)	17.9 (2.7)	10.4 (0.2)	3.7 (0.1)	33.6 (0.7)	5.3 (0.5)	55.2 (5.5)	147 (16.2)	7.4 (1.3)	6.5 (0.6)	21.5 (1.7)	43.6 (1.3)	31.9 (1.9)
3-Oxo-α-ionol	91.5 (11)	37.4 (2.2)	55 (6.1)	7.6 (1.1)	90.8 (8.2)	14 (1.8)	361.1 (57.8)	172.2 (15.5)	180.9 (31.2)	36.5 (4.8)	50.2 (3.5)	214 (23.5)	126.8 (2.5)
Vanillyl alcohol	193.9 (29.1)	1.8 (0.1)	36.2 (5.8)	13.1 (0.3)	67.1 (5.4)	14 (2.1)	469.8 (32.9)	157.1 (26.7)	131.9 (22.8)	45.2 (6.8)	81.5 (13.9)	364.8 (29.2)	126.4 (17.7)

nd: not detected.

**Table 2:** Variable of Importance for the Projection (VIP) score, statistical significance, level of significance (*p* < 0.1 *; *p* < 0.05 **; *p* < 0.01 ***).

	VIP Component 1	*p*-Value	Sign
*cis*-3-Hexen-1-ol	2.0616	0.01	***
Methionol	2.0096	0.032	**
2-Phenylethyl alcohol	1.9485	0.022	**
Bound 2,6-Dimethoxy phenol	1.8326	0.01	***
Bound *cis*-Linaloloxide	1.814	0.022	**
*trans*-Linaloloxide	1.7723	0.024	**
α-Terpineol	1.7117	0.046	**
Bound 3-Oxo-α-ionol	1.6867	0.046	**
∑ of Terpenes	1.6739	0.046	**
Geraniol	1.6189	0.063	*
Benzyl alcohol	1.5696	0.086	*
Bound 2-Phenylethyl alcohol	1.5631	0.063	*
1-Butanol	1.5193	0.086	*
Bound Vanillyl alcohol	1.4933	0.032	**
*cis*-2-Hexen-1-ol	1.4775	0.116	
Ethyl vanillate	1.4483	0.198	
3-Carene	1.4357	0.252	
Limonene	1.4263	0.1	*
Methyl vanillate	1.4196	0.032	**
β-Damascenone	1.396	0.022	**
Linalool	1.3485	0.063	*
Bound Methyl vanillate	1.3406	0.153	
α-Ionone	1.3361	0.199	
Bound α-Ionol	1.3297	0.152	
Bound *trans*-Linalooloxide	1.3033	0.116	
1-Hexanol	1.2344	0.253	
Terpinolene	1.1962	0.199	
DMTS	1.1892	nc	
Bound 3-Hydroxy-β-damascone	1.1848	0.153	
Hexanoic acid	1.184	0.253	
Carbon disulfide	1.1347	0.116	
Bound Ethyl vanillate	1.1087	0.153	
Methyl salycilate	1.1044	0.063	*
∑ of VSC	1.095	0.153	
∑ of Acids	1.0874	0.253	
Bound α-Terpineol	1.0837	0.199	
Isoamyl acetate	1.0804	0.391	
∑ of C6 alcohols	1.0526	0.475	
2,6-Dimethoxy phenol	1.0507	0.664	
∑ of acetate esters	1.0459	0.317	
Bound Geraniol	1.0447	0.086	*
Benzaldehyde	1.0439	0.153	
3-Methylbutanoic acid	1.0202	0.253	

nc: not calculable.

**Table 3:** Concentration (µg/L) of free volatile compounds in Cluster 1 and Cluster 2 wine samples. Odor threshold, mean, standard deviation (SD), statistical significance, level of significance (*p* < 0.1 *; *p* < 0.05 **; *p* < 0.01 ***).

	Cluster 1			Cluster 2				
	Mean (µg/L)		SD	Mean (µg/L)		SD	*p*-Value	Level
Alcohols								
1-Butanol	75	±	29	53	±	26	0.234	
2-Butanol	1278	±	547	840	±	514	0.100	*
Isoamyl alcool	87,886	±	13,492	83,158	±	9204	0.628	
Phenylethyl alcohol	7689.2	±	2940.9	8998.3	±	3056.6	0.534	
Methionol	99	±	39	140	±	45	0.089	*
1-Pentanol	155	±	36	156	±	21	0.945	
∑ Alcohols	97,182	±	13,312	93,345	±	11,482	0.731	
C_6_ alcohols								
1-Hexanol	817.3	±	178.1	964.9	±	133.4	0.295	
*cis*-3-Hexen-1-ol	28.9	±	16.2	25.4	±	9.9	0.534	
*trans*-3-Hexen-1-ol	55.7	±	19.3	72.9	±	24.3	0.234	
*cis*-2-Hexen-1-ol	9	±	1	10	±	2	0.534	
∑ C6 alcohols	911	±	196	1073	±	117	0.097	*
Acetate esters								
Isoamyl acetate	876.7	±	526.5	496	±	342.1	0.295	
n-Hexyl acetate	50.8	±	35.3	32.2	±	20.1	0.628	
2-Phenethyl acetate	112	±	99	77	±	35	0.945	
∑ Acetate esters	1039	±	580	606	±	375	0.234	
Ethyl esters								
Ethyl butanoate	270.9	±	48.7	236.5	±	77.7	0.534	
Ethyl 2-methylbutyrate	6.9	±	4.6	3.2	±	1.6	0.138	
Ethyl 3-methylbutanoate	12	±	7	11	±	8	0.628	
Ethyl 3-hydroxybutanoate	126	±	31	119	±	20	0.836	
Ethyl hexanoate	700	±	102	581	±	168	0.234	
Ethyl octanoate	725	±	223	613	±	113	0.295	
Ethyl decanoate	121	±	56	118	±	49	0.945	
Ethyl lactate	7646	±	5819	5052	±	3785	0.445	
∑ Ethyl esters	9607	±	5554	6733	±	3953	0.295	
Acids								
3-Methylbutanoic acid	184	±	41	237	±	49	0.089	*
Hexanoic acid	4932	±	1272	4863	±	635	0.945	
Octanoic acid	8391	±	1180	8518	±	345	0.628	
∑ Acids	13,507	±	2411	13,618	±	873	0.731	
Terpenes								
1,4-Cineole	0.6	±	0.32	0.57	±	0.17	0.976	
Limonene	0.5	±	0.18	0.29	±	0.13	0.015	**
1,8-Cineole	0.18	±	0.16	0.14	±	0.07	0.945	
γ-Terpinen	1.79	±	1.8	1.54	±	1.91	0.628	
*p*-Cymene	0.53	±	0.13	0.44	±	0.15	0.423	
Terpinolene	0.21	±	0.07	0.14	±	0.06	0.073	*
Linalool	1.5	±	1.79	0.3	±	0.28	0.079	*
Terpinen-1-ol	2.28	±	3.31	2.16	±	2.22	0.945	
Terpinen-4-ol	0.9	±	0.23	0.76	±	0.25	0.534	
Ho-trienol	0.04	±	0.09	0.13	±	0.28	0.061	*
α-Terpineol	2.93	±	0.63	1.77	±	0.54	0.008	**
β-Citronellol	1.39	±	1.21	1.03	±	1.26	0.505	
Nerol	0.92	±	1.38	2.12	±	2.58	0.945	
Geraniol	1.29	±	0.97	0.56	±	0.52	0.087	*
3-Carene	0.27	±	0.15	0.09	±	0.09	0.024	**
α-Phellandrene	0.24	±	0.19	0.21	±	0.15	0.836	
α-Terpinen	0.05	±	0.04	0.01	±	0.02	0.097	*
β-Myrcene	0.01	±	0	0.02	±	0.04	0.204	
*cis*-Linalooloxide	2.91	±	1.22	2.97	±	2.87	0.731	
*trans*-Linalooloxide	3.57	±	1.72	2.7	±	1.75	0.534	
*p*-Menthane-1,8-diol	0.9	±	1.2	2.3	±	2.8	0.443	
∑ Terpenes	24.1	±	4.8	21.2	±	10.2	0.138	
Sesquiterpenes								
Nerolidol	24.89	±	21.22	22.52	±	16.43	0.836	
Bisabolol	0.6	±	0.02	0.55	±	0.12	0.469	
Farnesol	0	±	0	0	±	0	0.337	
∑ Sesquiterpenes	25.5	±	21.2	23.1	±	16.5	0.628	
Norisoprenoids								
β-Damascenone	0.95	±	0.48	0.41	±	0.1	0.013	**
Vitispirane 1	0.69	±	0.39	0.52	±	0.34	0.534	
Vitispirane 2	0.41	±	0.16	0.47	±	0.25	0.976	
TPB	0.06	±	0.04	0.08	±	0.02	0.720	
TDN	1.33	±	0.71	1.92	±	0.78	0.366	
α-Ionone	0.77	±	0.02	0.76	±	0.01	0.114	
α-Ionol	0.76	±	0.03	0.76	±	0.01	0.487	
3-Hydroxy-β-damascone	0.02	±	0.04	0.01	±	0.01	0.883	
3-Oxo-α-ionol	3.45	±	1.82	4.35	±	1.95	0.534	
∑ Norisoprenoids	7.34	±	1.37	8.27	±	1.95	0.534	
Volatile sulfur compounds (VSC)								
3-MH	0.1	±	0.1	0.1	±	0.1	0.534	
Carbon disulfide	88.55	±	24.49	92.83	±	25.06	0.731	
DMS	1.29	±	0.59	1.39	±	0.91	0.945	
DES	0.78	±	0.2	0.71	±	0.23	0.628	
DMDS	0.02	±	0.02	0.02	±	0.02	0.971	
Ethyl thioacetate	0.88	±	0.34	0.75	±	0.24	0.628	
DEDS	0.01	±	0.01	0.01	±	0.01	0.336	
DMTS	0	±	0	0	±	0	0.860	
∑ VSC	91.7	±	25	95.9	±	25.3	0.731	
Benzenoids								
Methyl salicylate	53.41	±	69.07	25.4	±	16.9	0.534	
4-Ethyl guaiacol	19.31	±	41.12	4.47	±	6.36	0.864	
4-Ethyl phenol	3.47	±	0.05	3.45	±	0.01	0.948	
2,6-Dimethoxyphenol	3.41	±	0.84	3.25	±	0.6	0.457	
Vanillin	5.26	±	4.78	6.42	±	5.64	0.908	
Methyl vanillate	7.85	±	1.55	7.8	±	2.06	0.836	
Ethyl vanillate	4.57	±	3.76	3.77	±	1.89	0.760	
Vanillyl alcohol	5.5	±	3.9	3.9	±	3.3	0.945	
Benzaldehyde	29	±	17	18	±	4	0.181	
Benzyl Alcohol	220.4	±	113.3	124	±	45.4	0.138	
∑ Benzenoids	131.5	±	87.4	76.6	±	20	0.445	
Furfural	28.5	±	28.2	23.1	±	20.6	0.731	

**Table 4:** Odour threshold (μg/L), aromatic series, and odour activity values of some volatile compounds of wine samples.

			L1	L4	L5	L6	V3	V5	V1	V2	V4	V6	V7	L2	L3
	Odor Threshold (µg/L)	Aromatic Series ^16^	Cluster 1	Cluster 1	Cluster 1	Cluster 1	Cluster 1	Cluster 1	Cluster 2	Cluster 2	Cluster 2	Cluster 2	Cluster 2	Cluster 2	Cluster 2
Ethyl butanoate	20 ^1^	a	12.68	12.52	12.65	16.63	10.43	16.34	11.58	13.97	14.84	16.66	6.62	6.71	12.40
Ethyl 3-metilbutanoate	3 ^1^	a	0.27	0.62	0.22	0.78	1.15	0.56	0.49	0.17	0.13	0.52	0.35	1.21	0.82
Isoamyl acetate	30 ^1^	a	31.74	42.07	54.86	9.78	11.34	25.54	16.14	7.60	12.95	35.19	11.87	3.40	28.58
Ethyl hexanoate	14 ^1^	a	45.84	53.86	52.58	60.37	39.23	48.34	39.38	47.71	38.43	50.34	40.50	18.54	55.67
n-Hexyl acetate	1500 ^2^	a	0.08	0.04	0.04	0.02	0.01	0.02	0.02	0.01	0.02	0.04	0.02	0.00	0.03
Ethyl lactate	154,000 ^2^	a	0.01	0.10	0.01	0.03	0.09	0.06	0.03	0.08	0.06	0.01	0.02	0.01	0.02
Ethyl octanoate	5 ^1^	a	197.92	118.20	148.07	178.75	73.00	153.72	127.54	160.28	111.16	130.38	112.48	87.09	129.58
Ethyl decanoate	200 ^1^	a	1.11	0.42	0.60	0.60	0.30	0.60	0.59	1.05	0.45	0.70	0.48	0.26	0.62
β-Damascenone	0.05 ^1^	b	18.20	35.30	17.80	12.80	7.20	22.50	5.90	9.50	8.60	6.10	6.60	10.20	10.20
α-Ionone	2.6 ^3^	b	0.31	0.30	0.29	0.30	0.29	0.29	0.29	0.29	0.30	0.29	0.29	0.29	0.29
3-MH	0.06 ^1^	c	1.97	0.05	0.68	1.92	6.07	0.00	2.48	0.60	0.47	3.30	0.17	4.97	4.83
Phenylethyl acetate	250 ^1^	d	1.19	0.33	0.33	0.10	0.19	0.54	0.54	0.18	0.38	0.36	0.35	0.11	0.25
Linalool	15 ^2^	d	0.33	0.08	0.11	0.06	0.00	0.02	0.00	0.05	0.00	0.04	0.03	0.00	0.02
Terpinen-4-ol	100 ^8^	d	0.01	0.01	0.01	0.01	0.01	0.01	0.01	0.01	0.01	0.01	0.00	0.00	0.01
α-Terpineol	250 ^3^	d	0.02	0.01	0.01	0.01	0.01	0.01	0.01	0.01	0.01	0.01	0.00	0.01	0.01
β- Citronellol	40 ^2^	d	0.06	0.02	0.06	0.00	0.00	0.06	0.03	0.00	0.04	0.01	0.00	0.01	0.09
Nerol	400 ^4^	d	0.00	0.00	0.00	0.00	0.00	0.01	0.02	0.00	0.00	0.00	0.00	0.01	0.01
Geraniol	30 ^1^	d	0.07	0.04	0.09	0.02	0.03	0.01	0.00	0.04	0.02	0.01	0.02	0.05	0.00
1,4-Cineole	0.54 ^13^	e	0.52	0.75	0.61	1.96	1.65	1.15	0.71	1.50	1.15	0.63	1.08	1.33	0.99
1,8-Cineole	1.1 ^13^	e	0.06	0.02	0.09	0.42	0.20	0.17	0.09	0.19	0.25	0.10	0.07	0.08	0.11
Methyl salicylate	50 ^11^	e	0.38	3.84	0.53	1.00	0.28	0.38	0.15	0.35	0.53	1.13	0.23	0.75	0.42
1-Butanol	150,000 ^2^	f	0.00	0.00	0.00	0.00	0.00	0.00	0.00	0.00	0.00	0.00	0.00	0.00	0.00
Isoamyl alcohol	30,000 ^1^	f	2.64	3.07	2.42	3.73	2.91	2.81	2.84	2.77	2.92	3.29	2.69	2.60	2.29
1-Pentanol	64,000 ^12^	f	0.00	0.00	0.00	0.00	0.00	0.00	0.00	0.00	0.00	0.00	0.00	0.00	0.00
2-Phenylethanol	14,000 ^1^	f	0.77	0.44	0.30	0.38	0.60	0.80	1.03	0.64	0.68	0.65	0.69	0.51	0.30
Methionol	1000 ^1^	f	0.10	0.10	0.07	0.08	0.17	0.08	0.17	0.10	0.16	0.18	0.19	0.09	0.08
3-Methylbutanoic acid	33 ^1^	g	7.47	6.22	4.94	3.76	5.68	5.36	9.25	5.85	7.13	6.84	8.41	7.85	4.92
Hexanoic acid	420 ^1^	g	14.96	10.05	11.44	15.81	8.09	10.11	10.49	11.99	12.82	9.72	12.63	13.46	9.93
Octanoic acid	500 ^1^	g	20.11	15.14	16.81	18.64	13.53	16.47	16.90	18.27	16.77	15.97	17.19	17.27	16.87
Vanillin	60 ^2^	h	0.00	0.18	0.11	0.00	0.06	0.17	0.31	0.04	0.08	0.08	0.06	0.11	0.07
Vanillyl alcohol	5000 ^6^	h	0.00	0.00	0.00	0.01	0.00	0.00	0.00	0.00	0.00	0.00	0.00	0.00	0.00
Methyl vanillate	3000 ^2^	h	0.00	0.00	0.00	0.00	0.00	0.00	0.00	0.00	0.00	0.00	0.00	0.00	0.00
Ethyl vanillate	990 ^2^	h	0.00	0.00	0.01	0.01	0.00	0.00	0.00	0.00	0.00	0.01	0.00	0.01	0.00
Benzaldehyde	2000 ^7^	h	0.01	0.03	0.02	0.01	0.01	0.01	0.01	0.01	0.01	0.01	0.01	0.01	0.01
Benzyl alcohol	200,000 ^7^	h	0.00	0.00	0.00	0.00	0.00	0.00	0.00	0.00	0.00	0.00	0.00	0.00	0.00
4-Etilguaiacol	33 ^1^	h	0.10	0.10	0.09	0.09	3.13	0.00	0.56	0.00	0.00	0.09	0.10	0.10	0.09
2,6-Dimethoxy phenol	1850 ^7^	h	0.00	0.00	0.00	0.00	0.00	0.00	0.00	0.00	0.00	0.00	0.00	0.00	0.00
1-Hexanol	8000 ^1^	i	0.09	0.09	0.08	0.14	0.12	0.09	0.12	0.12	0.14	0.13	0.13	0.13	0.09
*trans*-3-Hexenol	1000 ^5^	i	0.03	0.05	0.06	0.07	0.08	0.05	0.05	0.08	0.10	0.05	0.05	0.07	0.11
*cis*-3-Hexenol	400 ^1^	i	0.06	0.07	0.12	0.11	0.05	0.02	0.05	0.05	0.05	0.07	0.04	0.07	0.11
*cis*-2-Hexenol	400 ^4^	i	0.03	0.03	0.02	0.02	0.02	0.02	0.02	0.03	0.02	0.02	0.02	0.02	0.03
DMS	10 ^1^	l	0.17	0.11	0.10	0.20	0.17	0.04	0.02	0.31	0.14	0.07	0.12	0.17	0.15
DES	18 ^15^	l	0.07	0.04	0.03	0.04	0.04	0.04	0.03	0.06	0.03	0.05	0.04	0.02	0.04
DMDS	29 ^14^	l	0.00	0.00	0.00	0.00	0.00	0.00	0.00	0.00	0.00	0.00	0.00	0.00	0.00
DEDS	4.3 ^14^	l	0.00	0.00	0.01	0.01	0.00	0.00	0.00	0.00	0.00	0.00	0.00	0.00	0.00
TPB	0.04 ^9^	m	1.13	2.38	1.38	0.00	1.75	2.88	1.38	2.75	1.75	1.13	1.75	2.25	2.13
TDN	2 ^10^	m	0.44	0.75	0.81	0.07	1.07	0.85	0.65	0.83	1.04	0.48	0.80	1.56	1.37

^1^ [45], ^2^ [71], ^3^ [72], ^4^ [73], ^5^ [74], ^6^ [75], ^7^ [76], ^8^ [77], ^9^ [78], ^10^ [79], ^11^ [28], ^12^ [80], ^13^ [31], ^14^ [81], ^15^ [82] ^16^ (a) fruity, (b) stewed apple/quince, (c) grapefruit, (d) floral, (e) fresh/minty, (f) vinous, (g) fermentative, (h) spicy, (i) green, (l) reductive, (m) evolutive.

**Table 5:** Aromatic series values for each samples, and Mann-Whitney test between clusters. Significant values (α = 0.1) are in bold.

Sample:	L1	L4	L5	L6	V3	V5	L2	L3	V1	V2	V4	V6	V7	Cluster Effect
Cluster:	1	1	1	1	1	1	2	2	2	2	2	2	2	*p*-Value
	298	235	277	275	143	253	125	235	203	238	186	241	180	**0.063**
Stewed apple/quince	18.20	35.30	17.80	12.80	7.20	22.50	10.20	10.20	5.90	9.50	8.60	6.10	6.60	**0.015**
Grapefruit	1.97	0.05	0.68	1.92	6.07	0.00	4.97	4.83	2.48	0.60	0.47	3.30	0.17	0.475
Floral	0.49	0.15	0.27	0.09	0.04	0.11	0.08	0.13	0.06	0.09	0.07	0.06	0.05	**0.086**
Fresh/Minty	0.44	3.86	0.62	1.41	0.48	0.55	0.84	0.53	0.24	0.54	0.78	1.22	0.30	0.475
Vinous	3.43	3.52	2.73	4.11	3.54	3.62	3.12	2.60	3.89	3.42	3.62	3.96	3.40	0.668
Fermentative	42.5	31.4	33.2	38.2	27.3	31.9	38.6	31.7	36.6	36.1	36.7	32.5	38.2	0.391
Spicy	0.11	0.32	0.24	0.12	3.22	0.19	0.23	0.18	0.89	0.06	0.09	0.20	0.18	0.391
Green	0.19	0.22	0.27	0.32	0.25	0.15	0.27	0.30	0.21	0.24	0.28	0.26	0.23	0.475
Reductive	0.47	0.33	0.40	0.47	0.35	0.20	0.34	0.34	0.22	0.54	0.28	0.30	0.39	0.475
Evolutive	1.88	3.42	2.48	0.37	3.10	4.02	2.32	3.87	3.08	1.89	2.83	4.10	3.78	0.534

**Table 6:** Wine samples and base enological parameters.

Code	Type	Vintage	pH		Ethanol (*v/v* %)		Free SO_2_ mg/L		Total SO_2_ mg/L		Tartaric Acid g/L	
L1	Lugana	2018	3.45	±0.01	13	±0.5	16.3	±0.8	79.2	±3.2	2.65	±0.04
L2	Lugana	2016	2.98	±0.02	12.5	±0.5	9.0	±0.5	62.0	±1.9	2.85	±0.11
L3	Lugana	2017	3.1	±0.02	12.5	±0.5	14.1	±0.8	97.5	±0.9	2.77	±0.11
L4	Lugana	2017	3.16	±0.01	12.5	±0.5	10.3	±0.5	77.9	±2.3	3.34	±0.02
L5	Lugana	2018	3.27	±0.01	12.5	±0.5	19.8	±0.2	134.5	±2.4	2.91	±0.02
L6	Lugana	2016	3.18	±0.03	13.0	±0.5	11.8	±0.1	44.5	±2.2	1.96	±0.08
V1	Verdicchio	2017	3.27	±0.01	13.5	±0.5	17.4	±0.5	70.0	±0.7	3.04	±0.08
V2	Verdicchio	2016	3.4	±0.01	13.0	±0.5	10.1	±0.2	84.0	±1.4	2.26	±0.09
V3	Verdicchio	2017	3.22	±0.03	12.5	±0.5	20.7	±1.0	97.5	±1.9	3.28	±0.11
V4	Verdicchio	2017	3.27	±0.03	12.5	±0.5	20.7	±1.0	84.0	±0.8	3.55	±0.14
V5	Verdicchio	2017	3.07	±0.01	13.0	±0.5	8.5	±0.4	41.5	±2.5	3.09	±0.04
V6	Verdicchio	2018	3.38	±0.02	12.5	±0.5	23.1	±0.5	93.5	±1.7	3.85	±0.15
V7	Verdicchio	2017	3.25	±0.01	12.0	±0.5	22.95	±1.4	92	±0.5	3.55	±0.09

**Table 7:** Retention indices, quantification ions of studied compounds.

	Method ^1^	LRI ^1^	Identification ^2^	Quantitation Ion m/z	Qualifier Ions m/z	LOD (µg/L)	LOQ (µg/L)
1-Butanol	a	1159	RS	56	55	0.02	0.06
2-Butanol	a	1020	RS	59		0.20	0.6
1-Pentanol	a	1256	RS	55	56, 57, 70	0.04	0.11
Isoamyl alcohol	a	1220	RS	57	55, 56, 70	0.02	0.06
Methionol	a	1710	RS	106	88, 73, 61	2.1	6.3
Phenylethyl Alcohols	a	1920	RS	91	65, 92, 122	1.95	5.84
1-Hexanol	a	1316	RS	56	55, 69	0.76	2.27
*trans*-3-Hexen-1-ol	a	1379	RS	67	55, 69, 82	0.40	1.21
*cis*-3-Hexen-1-ol	a	1391	RS	68	55, 69, 83	1.23	3.68
*cis*-2-Hexen-1-ol	a	1431	RS	82	71, 67	0.12	0.36
Isoamyl acetate	a	1125	RS	70	55, 60, 87	0.03	0.1
n-Hexyl acetate	a	1271	RS	56	55, 61, 84	0.03	0.1
2-Phenethyl acetate	a	1810	RS	104	91, 78	0.10	0.30
Ethyl 3-methyl butanoate	a	1069	RS	88	57, 60, 85	0.30	0.9
Ethyl butanoate	a	1032	RS	71	88	0.01	0.04
Ethyl hexanoate	a	1240	RS	88	60, 99	5.82	17.47
Ethyl octanoate	a	1430	RS	88	57, 100, 127	0.54	1.63
Ethyl decanoate	a	1640	RS	88	71, 101, 155	0.16	0.49
Ethyl lactate	a	1340	RS	75	88, 90	2.1	6.3
3-Methylbutanoic acid	a	1667	RS	60	87	0.17	0.52
Hexanoic acid	a	1839	RS	60	73, 87	0.15	0.46
Octanoic acid	a	2071	RS	60	73, 101, 115	0.00	0.01
*cis*-Linalooloxide	b	1437	RS	59	111, 94	0.02	0.07
*trans*-Linalooloxide	b	1469	RS	59	111, 94	0.02	0.07
Linalool	b	1547	RS	71	121, 93	0.01	0.03
Geraniol	b	1860	RS	93	123, 121, 105	0.02	0.07
β-Citronellol	b	1771	RS	69	82, 81, 67	0.07	0.21
α-Terpineol	b	1701	RS	136	121, 93, 59	0.23	0.7
α-Phellandrene	b	1180	RS	93	136, 91	0.001	0.003
Myrcene	b	1160	RS	93	79, 69	0.001	0.003
*p*-Cymene	b	1271	RS	119	134, 91	0.08	0.24
3-Carene	b	1130	RS	93	121, 91	0.01	0.03
α-Terpinen	b	1190	RS	121	136, 93	0.01	0.03
γ-Terpinen	b	1188	RS	121	93, 126	0.03	0.1
Limonene	b	1198	RS	136	139, 125, 111	0.02	0.08
1,4-Cineole	b	1186	RS	154	139, 111, 108	0.003	0.011
1,8-Cineole	b	1217	RS	154	139, 111, 108	0.003	0.011
*p*-Cymene	b	1271	RS	119	134, 91	0.02	0.06
Terpinolene	b	1283	RS	121	136, 93	0.03	0.09
Terpinen-1-ol	b	1581	LRI MS	136	121, 81	-	-
Terpinen-4-ol	b	1614	RS	71	111, 93, 86	0.02	0.05
*p*-Menthane-1,8-diol	a	2250	RS	96	88, 139	0.03	0.09
Ho-trienol	b	1585	LRI MS	82	67, 71	-	-
Nerol	b	1812	RS	93	121, 84, 69	0.04	0.12
β-Damascenone	b	1825	RS	69	190, 121, 105	0.01	0.03
α-Ionone	b	1853	RS	121	136, 192	0.02	0.06
α-Ionol	b	1925	RS	95	123, 138	0.04	0.12
3-Oxo-α-ionol	a	2555	LRI MS	108	152	-	-
3-Hydroxy-β-damascone	a	2532	LRI MS	175	208, 193	-	-
Vitispirane 1	b	1523	LRI MS	192	177, 93	-	-
Vitispirane 2	b	1529	LRI MS	192	177, 93	-	-
TPB	b	1828	LRI MS	172	157, 142	-	-
TDN	b	1745	LRI MS	157	172, 142	-	-
Nerolidol	b	2024	RS	69	161, 136, 93	0.015	0.05
Farnesol	b	2300	RS	69	136, 93, 81	0.03	0.1
Bisabolol	b	2206	RS	204	119, 109	0.03	0.1
Benzyl Alcohols	a	1874	RS	106	105, 77, 51	0.03	0.1
Vanillin	a	2572	RS	151	81, 152, 109	0.01	0.02
Vanillyl alcohol	a	2781	RS	154	137, 93	0.3	0.9
Furfural	a	1461	RS	95	96, 67	0.3	0.9
4-Ethyl guaiacol	a	1988	RS	137	122, 152	0.03	0.09
4-Ethyl phenol	a	2212	RS	150	107, 135	0.07	0.21
Ethyl vanillate	a	2665	RS	151	168, 196	2.36	7.09
Methyl salicylate	a	1763	RS	120	152, 92	0.45	1.35
Methyl vanillate	a	2630	RS	151	123, 182	0.97	2.91
Benzaldehyde	a	1500	RS	106	105, 77	0.87	2.61
2,6-Dimethoxyphenol	a	2270	RS	154	95, 111, 139	0.01	0.03
3-MH-ETP	c	2670	RS	232	187, 141	0.05	10
Carbon disulfide	d	760	RS	76	78	0.01	0.03
DMS	d	770	RS	62	61, 47	0.02	0.06
DES	d	910	RS	90	75, 61,62	0.03	0.09
DMDS	d	1075	RS	94	79, 64	0.09	0.27
Ethyl thioacetate	d	1090	RS	104	62, 60	0.03	0.09
DEDS	d	1180	RS	122	94, 66	0.06	0.18
DMTS	d	1375	RS	126	111, 79	0.07	0.21

^1^ Method: a (SPE for major compounds), b (SPME method for minor compounds), c (ETP derivatization followed by SPE extraction), d (SPME method for volatile sulfur compounds) ^2^ Linear Retention Index (LRI) were determined on DB-WAX polar column, as described by van Den Dool and Kratz (1963) [85]. RS identified using reference standard; LRI MS tentatively identified by comparing the Linear Retention Index and mass spectra with those of literature.

## Data Availability

The data presented in this study are available on request from the corresponding author.

## Supplementary Materials

The following are available, Figure S1: Total Ions Chromatogram (TIC) of V1 (Verdicchio) and L1 (Lugana) samples.

## Sample Availability

Samples of the compounds are available from the authors.
