# Supplementary figures and images for: Two Sides to One Story—Aroma Chemical and Sensory Signature of Lugana and Verdicchio Wines
Source: Molecules. 2021 Apr 7;26(8):2127. doi: 10.3390/molecules26082127 (PMC8067985; doi:10.3390/molecules26082127)

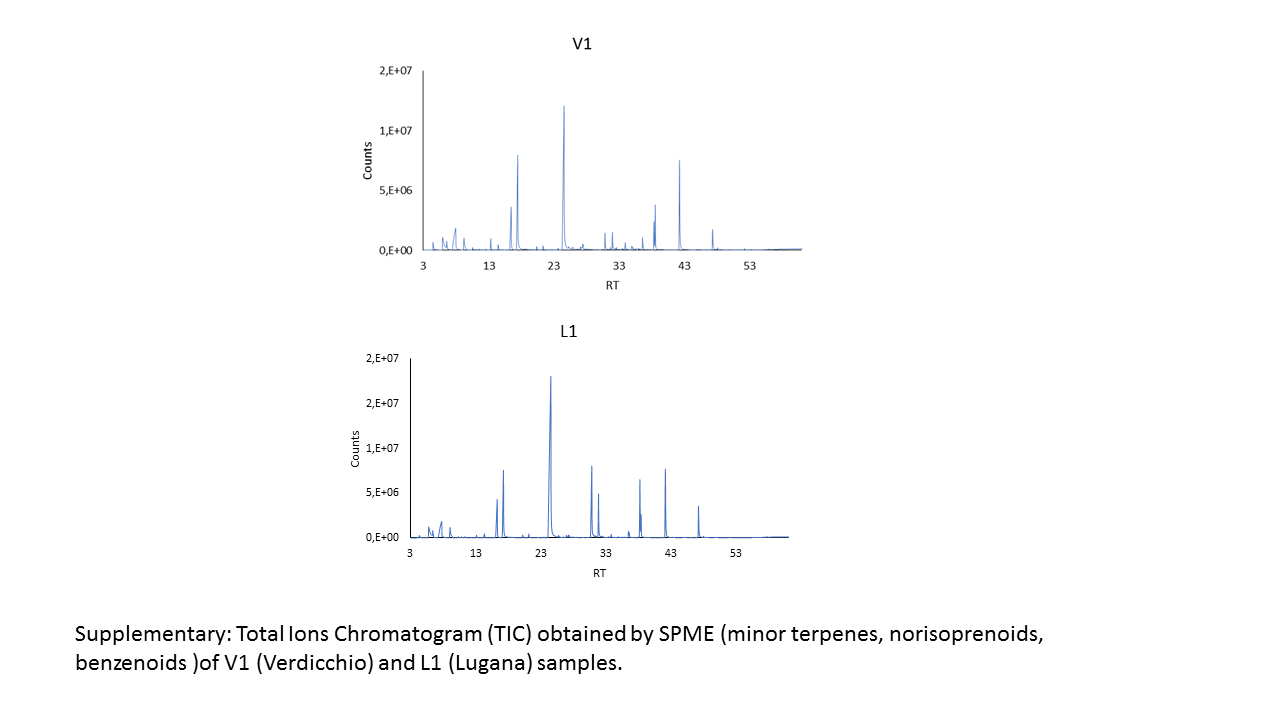

Supplement: Supplementary file 1 [file molecules-26-02127-s001.zip › molecules-1126801-supplementary.png]
